# Immobilization of α-transglucosidase on silica-coated magnetic nanoparticles and its application for production of isomaltooligosaccharide from the potato peel

**DOI:** 10.1038/s41598-023-38266-8

**Published:** 2023-08-05

**Authors:** Rohit Maurya, Usman Ali, Sunaina Kaul, Raja Bhaiyya, Ravindra Pal Singh, Koushik Mazumder

**Affiliations:** 1 National Agri-Food Biotechnology Institute (NABI), Sector-81 (Knowledge City), S.A.S. Nagar, Mohali, Punjab 140306 India; 2 Regional Centre for Biotechnology, Faridabad-Gurgaon, Haryana 121001 India; 3 Department of Industrial Biotechnology, Gujarat Biotechnology University, North Gate Gujarat International Finance Tech-City, Gandhinagar, Gujarat 382355 India

**Keywords:** Biochemistry, Biotechnology, Molecular biology, Chemistry, Nanoscience and technology

## Abstract

In this study, the production of isomaltooligosaccharide from potato peel starch was carried out in three steps: liquefaction, saccharification, and transglucosylation. Further, cloning α-transglucosidase gene from *Aspergillus niger* (GH31 family), transforming into *E. coli* BL21 (DE3), overexpressing and purifying the resulting protein for the production of α-transglucosidase. The generated α-transglucosidase was then bound with magnetic nanoparticles, which improved reusability up to 5 cycles with more than 60% activity. All the modifications were characterized using the following methods: Fourier transform infra-red analysis, Transmission Electron Microscopy, Field Emission Scanning Electron Microscopy, Energy Dispersive X-ray spectroscopy, X-Ray Diffraction Spectroscopy, Thermogravimetric Analysis, and Dynamic Light Scattering (DLS) analysis. Further, the optimum conditions for transglucosylation were determined by RSM as follows: enzyme-to-substrate ratio 6.9 U g^−1^, reaction time 9 h, temperature 45 °C, and pH 5.5 with a yield of 70 g l^−1^ (± 2.1). MALDI-TOF–MS analysis showed DP of the IMOs in ranges of 2–10. The detailed structural characterization of isomaltooligosaccharide by GC–MS and NMR suggested the α-(1 → 4) and α-(1 → 6)-D-Glcp residues as major constituents along with minor α-(1 → 2) and α-(1 → 3) -D-Glcp residues.

## Introduction

Isomaltooligosaccharides (IMOs) are non-digestible but fermentable oligosaccharides that increase the growth of certain health-beneficial bacteria, particularly *Bifidobacteria* and *lactobacilli*, which influence gut metabolism and have an impact on gastrointestinal microbial ecology^1–6^. IMOs are prebiotic oligosaccharides composed of glucose units linked by mainly α-(1 → 6) and α-(1 → 4) glycosidic linkage with a lower proportion of α-(1 → 3) (nigerose) and α-(1 → 2) (kojibiose) glycosidic linkage^7–9^. Typically, IMOs have different degrees of polymerization (DP) which include isomaltose (DP2), isomaltotriose (DP3), isopanose, panose (DP3), isomaltotetrose (DP4), and isomaltopentose (DP5)^10,11^. The IMOs are not digested by human enzymes but fermented by gut flora which exerts their prebiotic effect^5,12,13^. In addition to the prebiotic effect, IMOs have a low glycemic index and act as a low-calorie sweetener that provides health benefits to diabetic persons^10,14^.

Commercially, IMOs are produced from starch taken from various sources (sweet potato, potato, tapioca, rice, and banana)^11,15–18^. Generally, the traditional method employed for the production of IMOs consisted of three steps: liquefaction, saccharification, and transglucosylation^6,19^. Recent studies have demonstrated the simultaneous saccharification and transglucosylation (SST) for the production of IMOs^16,20^. These studies improved the efficiency of IMOs production and decreased the reaction time. Different studies have also reported enzymatic production of IMOs from sucrose using dextransucrase and dextranase^21–23^. Further, it was reported that the development of magnetic nanoparticles (MNPs) based immobilization of α-transglucosidase is one best way to improve the reusability of the enzyme^24–26^.

In this study, the extraction of starch from potato peel and then liquefaction and saccharification of liquefied starch were first carried out. A gene encoding α-transglucosidase (average MW ~ 110 kDa) from *Aspergillus niger* (GH31 family) was synthesized by GenScript (Singapore) and cloned into the pET28a vector. Given the importance of enzymes for IMOs production, an effort was made to express heterologously in *E. coli* BL21(DE3). Further, the transglucosylation reaction was optimized by RSM to maximize the yield of IMOs. Thereafter, produced α-transglucosidase was immobilized with MNPs and characterized by various analytical techniques. Finally, the structural features of purified IMOs fraction were analysed by using MALDI-TOF–MS, GC–MS, and NMR.

## Materials and methods

### Materials

Potato peel waste was collected from the institute mess (NABI Mohali, Punjab, India) and used for starch extraction. All chemicals used in IMOs characterization and two enzymes, Termamyl® SC DS (A4862) and Fungamyl® (A8220), used for liquefaction and saccharification reaction were purchased by Sigma-Aldrich. The α-transglucosidase gene sequence of *Aspergillus niger* (GH31 family) was synthesized and cloned in the pET28a vector by GenScript Biotech Corporation. The standards glucose, maltose, maltotriose, isomaltose, isomaltotriose, panose, isopanose, and all other chemicals used in the present study were also purchased from Sigma-Aldrich.

### Starch extraction

Potato peels (100 g) were first washed and then blended using a laboratory-scale blender. Immediately, the blended mixture was filtered and the left residue rinsed with deionized water (5 × 200 ml) 2 to 3 times. The filtrate was collected into a beaker and kept at 4 °C for settled-down starch. The supernatant was discarded and the white layer of starch was collected from the beaker into an oven tray and allowed to dry in a hot air drier at 37 °C for 24 h. Following this, dried residues were grounded in fine powder and stored in an airtight container for later use^27,28^.

### Liquefaction

#### Effect of enzyme-to-substrate ratio

Extracted starch was added to water and made a slurry(1 g 10 ml^-1^). The pH of the slurry was fixed at 6.9 with lactic acid. Different ratios of an enzyme (Termamyl® SC DS) to substrate (starch) such as 0.3–5.5 U g^-1^ were used. The mixer was placed at 95 °C for 1 h. The residual starch was estimated with the iodine (KI/I_2_) test^29^.

#### Effect of pH

The starch slurry (1 g.10 ml^-1^) was adjusted at different pH (4–8). The slurry was liquefied with a fixed enzyme (Termamyl® SC DS) to substrate ratio (0.7 U g^-1^) at a fixed temperature of 95 °C for 1 h. The residual starch was estimated as mentioned above.

#### Effect of temperature

Initially, 1 g.10 ml^-1^ starch was hydrolysed with a fixed enzyme (Termamyl® SC DS) to substrate ratio (0.7 U g^-1^) and pH (6.9) at different temperatures (65–110 °C) for 1 h^6,30,31^.

### Saccharification

#### Effect of enzyme-to-substrate ratio

The liquefied slurry was further saccharified with a different ratio of the enzyme (Fungamyl®) to substrate (0.7–9.6 U g^-1^) which affected the yield of maltooligosaccharide. The other three factors (temperature, pH, and time) were set at 50 °C, pH 5.5, and 12 h, respectively. After 12 h, the reaction was stopped, and TLC and HPAEC-PAD were used to analyse the reaction mixture^6,30,31^.

#### Effect of time

The effect of different reaction times (2–12 h) also affected the yield of maltooligosaccharide when the other three factors (enzyme to substrate ratio, temperature, and pH) were fixed at 1.2 U g^-1^, 50 °C, and pH 5.5, respectively. All reactions were stopped and analysed as mentioned above.

#### Effect of temperature

The liquefied slurry was saccharified with a fixed enzyme-to-substrate ratio (1.2 U g^-1^) and pH 5.5 at different temperatures (40–70 °C) for 4 h. After 4 h, reaction was stopped and analysed as mentioned above.

#### Effect of pH

The reaction mixture varied at different pH (4.5–7.5), affecting maltooligosaccharide yield when the other three factors (enzyme to substrate ratio, temperature, and time) were set at 1.2 U g^-1^, 50 °C, and 4 h, respectively. After 4 h, the reaction mixture was stopped by boiling mixture for 10 min and the reaction mixture was analysed as mentioned above.

### Production of α-transglucosidase

The synthesized α-transglucosidase gene was cloned in pET-28a and transformed into *E. coli* BL21(DE3). The enzyme was induced with IPTG (0.2 mM) overnight at 18 °C in 1 L terrific broth. Afterward, cells were harvested by centrifugation (7000 rpm, 10 min) and lysed by sonication in buffer A (10 mM 4-(2-hydroxy-ethyl)-1-piperazineethanesulfonic acid [HEPES]) and cells were resuspended in an appropriate buffer having protease inhibitors as well as lysed by sonication. Subsequently, the supernatant was collected, the enzyme was purified by nickel-nitrilotriacetic acid (Ni–NTA) purification column, and molecular weight was determined by Sodium dodecyl-sulfate polyacrylamide gel electrophoresis (SDS-PAGE)^32^.

#### Purification of α-transglucosidase

HisTrap HP column (GE Healthcare) was used to purify the α-transglucosidase protein. N’ terminal His6-tagged recombinant α-transglucosidase protein was put onto a column that had already been pre-equilibrated with buffer A (10 mM HEPES of pH 7.5, 250 mM NaCl). Buffer B (10 mM HEPES of pH 7.5, 250 mM NaCl, and 500 mM imidazole) was used to elute bound proteins in one step after washing with buffer A. Further α-transglucosidase protein was purified and concentrated with Amicon Ultra-15 100 kDa molecular weight cut-off concentrator. The purified α-transglucosidase was then kept at −80 °C for further usage^32^.

#### Secondary and Tertiary Structure characterization of α-transglucosidase protein

Circular dichroism (CD) spectroscopy was used to monitor the secondary structural alterations in free enzyme after immobilization. Biologic spectrometer MOS-500 equipment was used to record the UV CD spectra in the 190–280 nm wavelength region. The 0.2 mg/mL enzyme concentration was employed in all CD measurement experiments at 6 °C in a diluted buffer (Potassium phosphate buffer, pH 7.4). Tertiary structural model of α-transglucosidase was generated in Phyre 2 and processed in PyMOL software^33^. It was superimposed on a α-transglucosylase (protein data bank ID: 4B9Z) for identifying critical residues^34^.

### Transglucosylation

#### Effect of enzyme-to-substrate ratio

The saccharified slurry was further treated by the 0.7–11 U g^-1^ enzyme (α-transglucosidase) to substrate (saccharified starch) ratio at three fixed factors, temperature, pH, and time were 45 °C, 5.5, and 12 h, respectively. The product was analysed by TLC and High-performance Anion Exchange Chromatography pulsed amperometric detection (HPAEC-PAD)^1–6^.

#### Effect of time

The saccharified starch was incubated with a 5.6 U g^-1^ enzyme (α-transglucosidase) to substrate (saccharified starch) ratio at 45 °C and pH 5.5. Samples were drawn at different time intervals (2–12 h) for time optimization. The product was analysed as mentioned above.

#### Effect of pH

The effect of pH on the IMOs yield varied between 3.5 and 7.5 when enzyme to substrate ratio, temperature, and time were set at 5.6 U g^-1^, 45 °C for 6 h in a shaking water bath, respectively. After 6 h, the reaction was stopped and the product was analysed by TLC and HPAEC- PAD.

#### Effect of temperature

The saccharified starch was placed at a different temperature varying between 35 and 75 °C and enzyme to substrate ratio, pH, and time were set at 5.6 U g^-1^, 5.5 pH for 6 h, respectively. The reaction mixture was analysed as mentioned above.

### Experimental design for optimization of optimum condition for transglucosylation reaction and statistical analysis

The Box-Behnken was used to optimize the best condition for the transglucosylation reaction. The factors investigated in this study were enzyme-to-substrate ratio (A), slurry pH (B), temperature (°C), and reaction time (h). The three factors were evaluated at + 1, 0, and − 1 for high, intermediate, and low levels. The design contains a total of 29 runs based on Box-Behnken^35^.

### α-transglucosidase immobilization

#### ***Synthesis of magnetic nanoparticles (Fe***_***3***_***O***_***4***_***)***

The magnetic nanoparticles (Fe_3_O_4_) were synthesized by coprecipitation of ferric and ferrous chloride in the presence of 1.5 M NaOH as reductant. To prepare magnetic nanoparticles, solution 5.4 g FeCl_3_.6H_2_O was dissolved in 25 ml of 0.4 M HCl with 2 g FeCl_2_.4H_2_O and the reaction mixture was placed at 200 °C until a yellow transparent solution was formed. Further, the yellow transparent solution was dropwise added to 1.5 M NaOH solution at 80 °C. As the yellow solution was added to the NaOH solution, black precipitation of Fe_3_O_4_ formed. The precipitate was collected in a magnetic stand and washed with MQ water. The precipitate was stored in 200 ml of 0.1 M tetramethylammonium hydroxide (TMAOH) solution for further use^24,26^.

#### ***Synthesis of silica-coated magnetite nanoparticle Fe***_***3***_***O***_***4***_***@Si***

Synthesized *Fe*_*3*_*O*_*4*_ was stabilized by silica coating, which also prevented agglomeration from forming as a result of interparticle interaction. TMAOH stored magnetite was put in a falcon magnetic stand, and the supernatant was discarded. Magnetite was transferred to the beaker with a 1:1.4 volume ratio of ethanol and 10% tetraethyl orthosilicate (TEOS). The mixture was placed at 90 °C for 6 h for the synthesis of silica-coated nanoparticles, followed by washing and storage^24,26^.

#### ***Functionalization of Fe***_***3***_***O***_***4***_***@Si with 16-Phosphonohexadecanoic acid (16-PHDA) linker***

The obtained silica-coated magnetite nanoparticles were linked with a 16-PHDA linker in 1:1 ratio. The mixture was placed in an ultrasonicator for 30 min. After 30 min, the solution was rinsed with water and kept for future use^24,26^.

#### Immobilization of α-transglucosidase with 16-PHDA functionalized magnetite nanoparticle

In order to couple α-transglucosidase with the carboxylic groups of 16-PHDA, the mixture was first activated using EDC in 0.1 M MES buffer, then α-transglucosidase was added in an equal amount, and the mixture was left at room temperature for 2 h^24^. The Bradford method was used to calculate the amount of enzyme immobilized on nanoparticles in terms of protein contain. The following equation was used to determine the enzyme loading percentage:

$$Loading (\%)=\frac{Ei}{Et}\times 100$$

*Ei* = *Et–Es*, Ei = Immobilized enzyme, Et = Initial amount of added enzyme, Es = Amount of enzyme in supernatant.

### Characterization methods

Agilent Cary, 660 series with DTGS detector spectrophotometer, was used to record the IR spectra of the samples at frequencies between 400 and 4000 cm^−1^ ^26^. Transmission electron microscopy with high resolution (HR-TEM, LIBRA 120), 300 kV used for the size and shape of Fe_3_O_4_, Fe_3_O_4_@SiO_2_, Fe_3_O_4_@SiO_2_-16 PHDA, and Fe_3_O_4_@SiO_2_-16 PHDA-α-transglucosidase. The size and surface morphology of Fe_3_O_4_ and Fe_3_O_4_@SiO_2_ were examined using scanning microscopy (FE-SEM, Thermoscientific Apreo S)^24,26^. By using an X-ray diffractometer (XRD; Rigaku, Smart LAB SE), the purity and crystallinity of Fe_3_O_4_, Fe_3_O_4_@SiO_2_, Fe_3_O_4_@SiO_2_-16 PHDA, and Fe_3_O_4_@SiO_2_-16-PHDA-α-transglucosidase were measured^26,36^. Further, the percentage weight loss of samples was analyzed by using thermogravimetric analysis (TGA) (Netzsch simultaneous thermal analyser STA 449F1) at temperatures ranging from 25 to 800 °C in a nitrogen (N_2_) atmosphere. Finally, the zeta potential and colloidal stability were measured using Dynamic Light Scattering (DLS) analysis^24,26^.

#### Stability and reusability of immobilized enzyme

To determine the stability of free and immobilized α-transglucosidase at different pH and temperature conditions, the maltooligosaccharides were incubated with α-transglucosidase at pH values ranging from 3.5 to 8.5 and temperatures (30–80 °C), respectively. Finally, the percent relative activity in each experiments represents the enzyme activity relative to the control, which was assumed to be 100%^37,38^.

The reusability of the immobilized α-transglucosidase was analyzed by hydrolysis of maltose, under optimized conditions (enzyme to substrate ratio 6.9 U g^-1^, reaction time 9 h, temperature 45 °C, and pH 5.5). After reaction completion, immobilized α-transglucosidase was recovered by a magnetic stand at the end of each cycle, thoroughly cleaned two to three times with deionized water, and then used for the subsequent cycle of the reaction. The recovered immobilized α-transglucosidase was reused in the fresh maltose solution. The first cycle of the immobilized enzyme's activity was considered the control, with 100% activity^24^.

#### Determination of kinetic parameters

The optimized assay condition was used to establish the kinetic parameters of free and immobilized α-transglucosidase, with the exception that the maltose concentration was changed from 100 to 500 mM. Michaelis–Menten and Lineweaver–Burk plots were used to calculate the kinetic parameters, including the Michaelis–Menten constant (Km), turnover number (Kcat), and catalytic efficiency (Kcat/Km)^39^.

#### Purification of IMOs

The crude IMOs were purified with HPLC-RI on a superdex peptide 10/300 column (10 × 300–310 mm). The sample was put in the chromatography cabinet at 40 °C. The autosampler was used to inject the sample (15 µl) into the column. The sample was eluted at a flow rate of 0.5 ml/min with deionized distilled water and the effluent was manually collected. Further, the purified sample was characterized by TLC, HPAEC-PAD, MALDI-TOF–MS, GC–MC, and NMR^40^.

#### Thin layer chromatography

IMOs samples (5 mg ml^-1^) were spotted at a distance of 1 cm from the bottom on TLC silica gel glass plates (60 F 254, 10 × 20 cm; TLC silica gel, Sigma Aldrich). After spotting samples were allowed to dry. The bottom part of the plate was submerged in the mobile phase (n-propanol/water/ethanol in 70:20:10 v/v/v). The sample spots drifted upward direction with the mobile phase. The plates were dried when the mobile phase reached the second end of the plate. The spraying solution (5gL^-1^α-naphthol and 50 mL L^-1^ H_2_SO_4_) was applied on the TLC plates. The plates were heated at 120 °C for 10 min^18^.

#### MALDI-TOF–MS

The MALDI-TOF–MS (AB SCIEX 5800) was used to record the mass spectrum of the purified IMOs sample. An equivalent volume of matrix solution (0.1 M 2,5-dihydroxybenzoic acid and 0.03 M 1-hydroxyisoquinoline in aq. 50 percent ACN) was mixed with an aqueous sample. The sample mixture was loaded on a MALDI target plate and dried^41^. A mass spectrum was generated by using the spectra from 200 laser pulses^42^.

#### Linkage analysis

To investigate the glycosyl linkage composition of IMOs, partially methylated alditol acetate derivatives were produced. The IMOs samples (5 mg) and 15 mg NaOH powder were added in dry DMSO. Each sample received 1 ml of iodomethane, and the mixtures were mixed for 30 min at room temperature. A nitrogen stream was used to remove excess iodomethane, and samples were divided into two layers: upper (water) and bottom (chloroform). The bottom layer (chloroform) was recovered and dried and stored for analysis. The derivatized samples were then analyzed using GC-FID-MS^35,42^.

#### NMR

The purified IMOs (10 mg) were dissolved in 0.5 ml D_2_O. A 600 MHz NMR spectrometer was used to generate ^1^H NMR spectra. Every 2D NMR spectrum was recorded with standard Bruker pulse techniques. The internal acetone standard was used to measure chemical changes^41,42^.

#### Statistical analysis

All the experiments were repeated in triplicate and analyzed by the Analysis of variance (ANOVA) test using Graph Pad 6.0 and Prism 5^43^. The results of statistical analysis were expressed as mean ± SEM. Differences between the mean values of the measured properties were compared using multiple-range Tukey's test. In all cases, a *p*-value less than 0.05 was statistically significant^44^.

## Results and discussion

### Liquefaction and saccharification of starch extracted from potato peel

In the present study, starch from potato peel was used for IMOs production. Starch was isolated from wet potato peel with a yield of 6.5% (± 1.1). IMOs were produced using three enzymatic pathways: liquefaction, saccharification, and transglucosylation. In the first step, the optimum condition for nearly complete liquefaction of potato starch was determined as time: 1 h, temperature: 95 °C, pH: 6.9, and enzyme (Termamyl®) to substrate ratio: 0.7 U g^-1^. The completion of liquefaction was identified by the dispersion of color of the starch iodine complex during the reaction. Further, in the second step, Fungamyl® was used for the saccharification. The optimum condition for complete saccharification of liquefied starch was determined as time: 4 h, temperature: 50 °C, pH: 6–6.5, and enzyme to substrate ratio: 1.2 U g^-1^. The HPAEC-PAD analysis of the saccharified product showed the presence of maltose (72 g l^-1^), and maltotriose (18 g l^-1^) as major constituents along with a small amount of glucose (13 g l^-1^). The detailed explanation is provided in the supplementary data (Supplementary Fig. S1). The optimized condition of liquefaction and saccharification was found to be similar, as reported in the previous studies^6,16^.

### Production of α-transglucosidase

**Figure 1:**
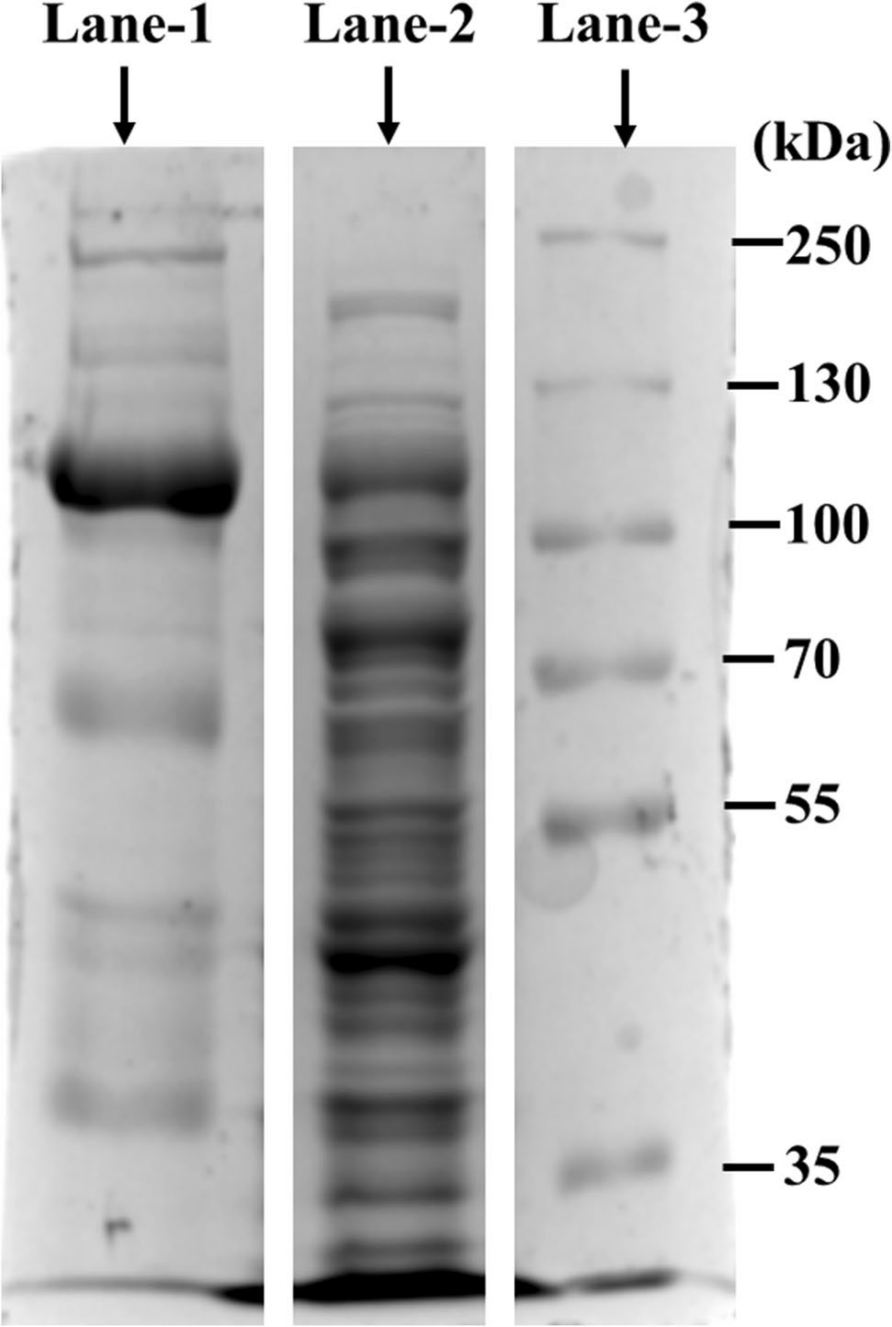
10% SDS PAGE analysis for estimation of molecular weight of produced α- transglucosidase. (Lane 1 purified α-transglucosidase; lane 2 Crude enzyme (0.2 IPTG); lane 3 protein marker). The SDS PAGE display image cropped from the original image presented in Supplementary Fig. S2.

The gene (α-transglucosidase) from *A. niger* was cloned in the pET-28a vector (GenScript Biotech Corporation) and further overexpressed. The purified α transglucosidase was analyzed on SDS-PAGE, indicating a molecular weight of about 110 kDa. The display image of SDS-PAGE (Fig. 1) cropped from the original image which is presented in Supplementary Fig. S2. In a previous study α-transglucosidase encoding gene from *A. niger* was cloned and expressed in *Pichia pastoris* to produce α-transglucosidase^45^. However, it was cloned and produced in *E. coli* in the current study for the first time.

### Secondary and tertiary structure analysis of α-transglucosidase

**Figure 2:**
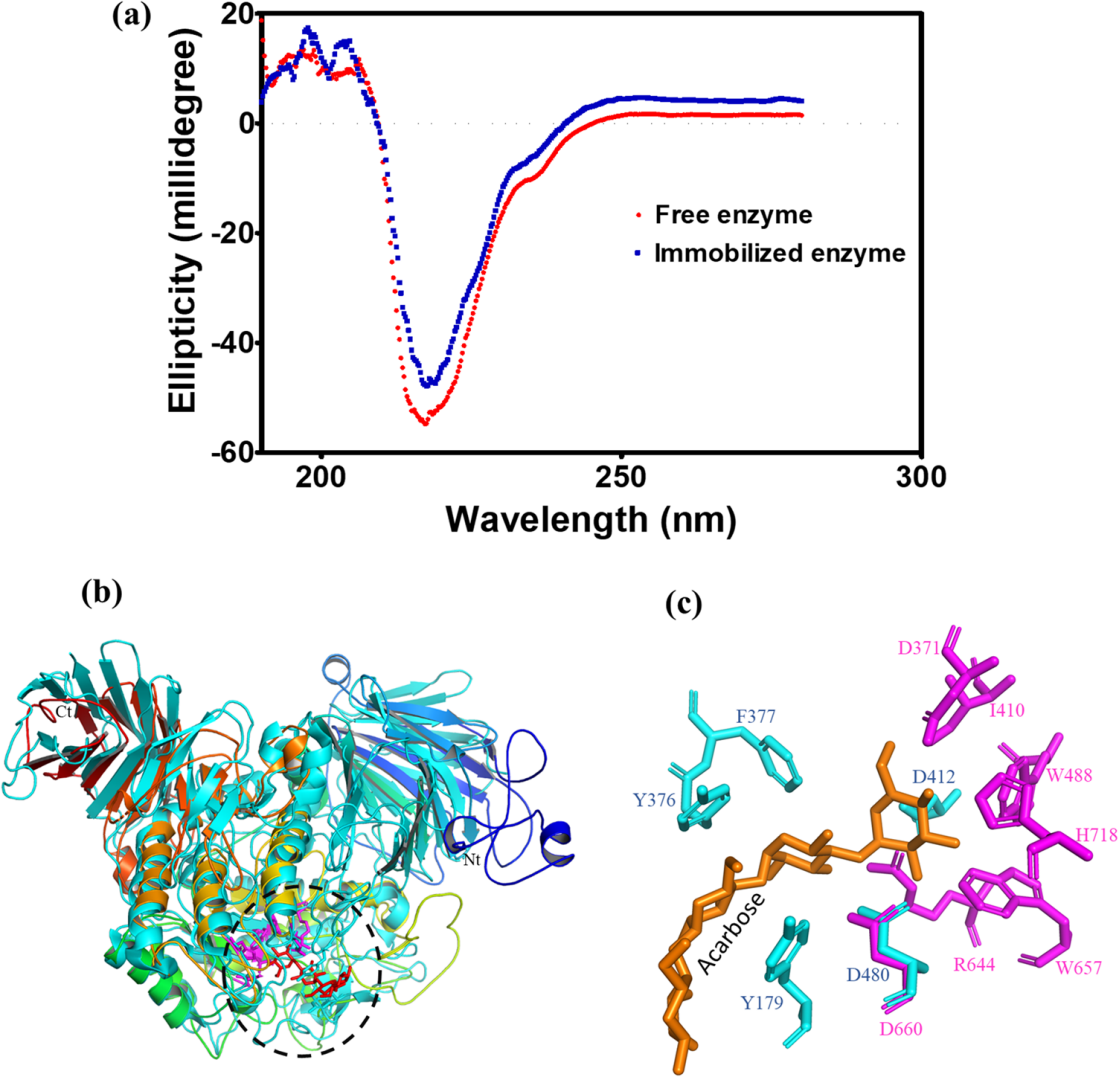
Secondary and tertiary analysis (**a**). CD spectrum of Immobilized magnetic nanoparticles (MNPs) with α-transglucosidase, and free α-transglucosidase; (**b**). Tertiary structural model of α-transglucosidase was generated in Phyre 2 and processed in PyMOL software. It was superimposed on a α-transglucosylase (protein data bank ID: 4B9Z) for identifying critical residues; (**c**). Crystal structure of α-transglucosylase (4B9Z) suggested that substrate-binding pocket of α-transglucosidase made up of narrow groove with residues of D371, I410, W488, H718, R644, W657, and D660.

CD spectra of free and immobilized α-transglucosidase were studied to analyse the impact of immobilization on the enzyme's secondary structure. Overall, there are no changes in secondary structures of free and immobilized α-transglucosidase (Fig. 2a), suggesting that current immobilized technique does not interfere with the structure of α-transglucosidase which was also in agreement with the previous study^34^.

The tertiary structure of α-transglucosidase (4B9Z) suggested that substrate-binding pocket of α-transglucosidase made up of narrow groove with residues of D371, I410, W488, H718, R644, W657, and D660 (Fig. 2b and c). In the current study, D371 and D660 are likely to act as nucleophilic attackers and acid–base residues of α-transglucosidase. These were revealed through superimposing a crystal structure of α-transglucosylase (4B9Z) on a model of α-transglucosylase whose D412 and D480 function as a nucleophilic attacker and an acid–base residue, respectively. The Asp660 is likely to catalyse maltooligosaccharide by breaking α-(1,4) glycosidic linkages and transferring glucose on maltose or glucose.

### Optimization of α- transglucosylation of saccharified starch

In order to determine the optimum transglucosylation reaction for the production of IMOs, the effect of four variables enzyme-to-substrate ratio, temperature, pH, and time were investigated. The single-factor experimental data revealed that the highest yield of IMOs (58 g l^-1^) with glucose and maltose as other constituents were plotted by HPAEC-PAD data (supplementary Fig. S3) under the optimum condition such as enzyme-to-substrate ratio: 5.6 U g^-1^, temperature: 45 °C, pH: 5.5, and time 6 h^1–6^.

**Figure 3:**
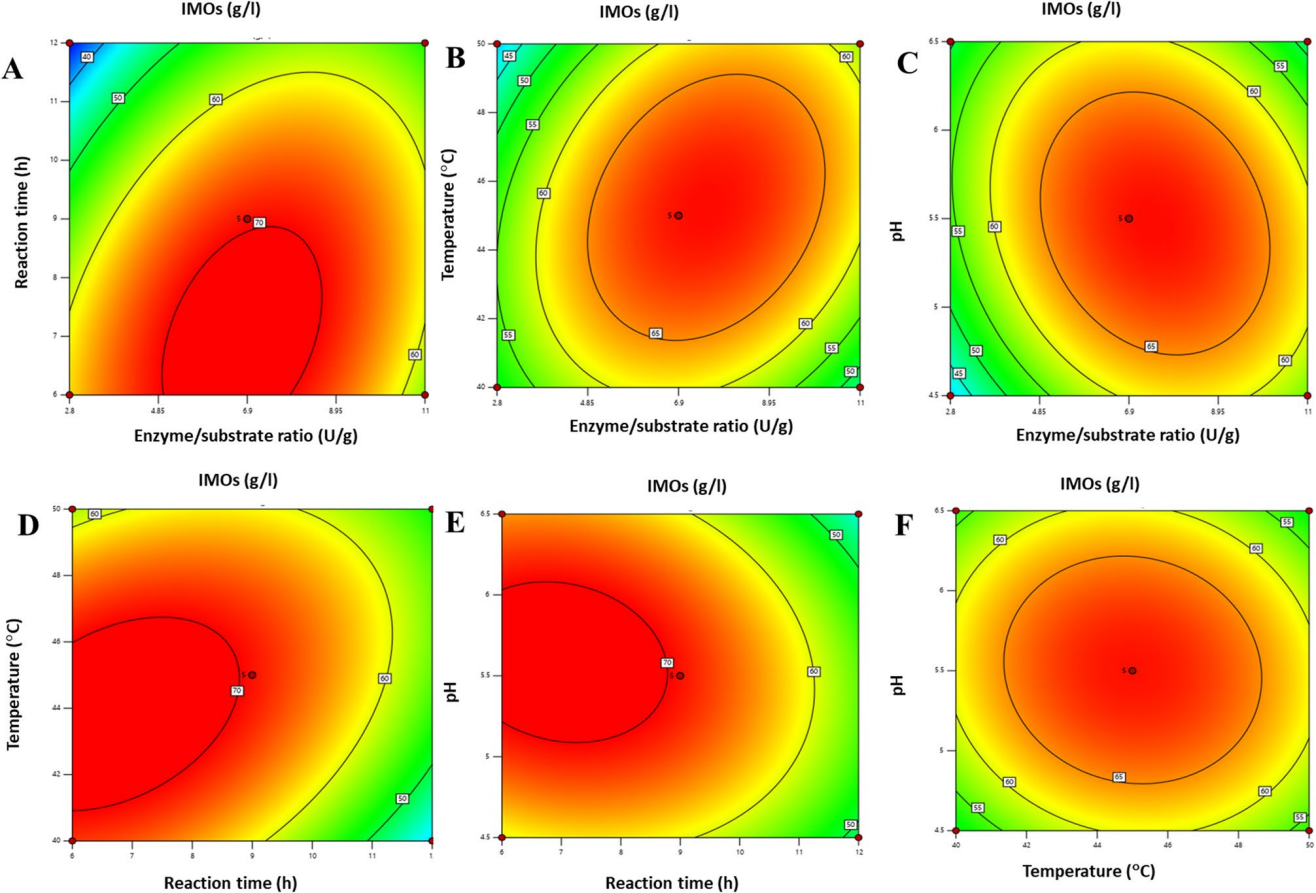
Contour plots for the effect of variable parameters on the yield of IMOs, A. Reaction time and Enzyme/substrate ratio, B. Temperature and Enzyme/substrate ratio, C. pH and Enzyme/substrate ratio, D. Temperature and reaction time, E. pH and Reaction time, F. pH and Temperature.

The Box-Behnken experimental design was employed to study optimum conditions to achieve the highest IMOs yield. The relationship between the independent and dependent variables can be expressed by yield: Y(IMOs) = 70.00–5.29A + 5.24.1B D-1.18 CD-5.76A^2^-2.23B^2^ + 0.72 C^2^-1.33D^2^. The IMOs yield data is shown in Table 1. ANOVA was used to determine the sufficiency and fitness of the impacts of independent factors on the yield (supplementary Table S1). The proposed model had a low *P* value (0.0001), showing a highly significant model. The adjusted R^2^ of determination, which was 0.9742, also indicated that this model was highly significant. The outcome showed that the IMOs yield was significantly affected by all independent (A, B, C) and quadratic factors (A2, B2, C2)^35^. The response surface plots in Fig. 3 showed the effects of independent variables and how they interacted with one another on the yield of IMOs. After transglucosylation the products were analyzed by HPAEC-PAD. The HPAEC-PAD estimated the highest yield of IMOs as 70 g l^-1^ (± 2.1) at a condition developed by the RSM model, which was enzyme to substrate ratio 6.9 U g^-1^, reaction time 9 h, temperature 45 °C, and pH 5.5.

**Table 1:** Box-Behnken for the optimization of process for IMOs production from saccharified starch using response surface methodology.

Run	Enzyme/substrate ratio (U g^-1^)	Time (Hours)	Temperature (°C)	pH	Isomaltose (g l^-1^)	Isomaltotriose (g l^-1^)	Panose (g l^-1^)	IMOs* (g l^-1^)
1	6.9	9	40	4.5	27.6	8.2	1.5	50
2	6.9	6	45	4.5	20	4.2	33.6	61
3	6.9	12	40	5.5	23	8	0	39
4	6.9	6	40	5.5	31.6	14.4	4.1	66
5	2.8	6	45	5.5	15	2.4	42	63
6	6.9	12	50	5.5	34	14	0	54
7	6.9	9	45	5.5	35.4	13.6	11.7	68
8	6.9	9	40	6.5	21	7	21	52
9	6.9	9	45	5.5	35.4	13.6	11.7	70
10	6.9	12	45	6.5	25	7.2	16.1	45
11	6.9	6	45	6.5	29.5	8.6	15.4	64
12	2.8	9	45	4.5	12.5	2.4	22.2	39
13	6.9	9	45	5.5	35.4	13.6	11.7	67
14	6.9	9	45	5.5	33.5	12.6	13.2	69.3
15	6.9	9	45	5.5	32	13	11	68
16	6.9	12	45	4.5	26	9	16.1	50.4
17	11	12	45	5.5	32.6	13.4	0	51.3
18	6.9	9	50	4.5	24	9	9	54
19	2.8	12	45	5.5	15	6	0	34
20	11	9	50	5.5	32.5	12	0	55
21	2.8	9	50	5.5	12.5	2.4	22.2	39
22	6.9	6	50	5.5	20.6	4.3	28	59.9
23	11	9	45	4.5	32.5	12	0	56
24	11	9	45	6.5	32.5	12	0	50.5
25	2.8	9	40	5.5	11.3	2.3	40	55
26	2.8	9	45	6.5	11.3	2.3	40	49
27	11	9	40	5.5	32.5	12	0	50.5
28	6.9	9	50	6.5	23.6	7.4	8	51
29	11	6	45	5.5	32.5	12.4	11	56

*Total IMOs represents all isomaltooligosaccharide products including, kojibiose, nigerose, isopanose and DP > 3.

### Immobilization of α-transglucosidase

MNPs were used to immobilize the α-transglucosidase. FeCl_2_ and FeCl_3_ salts were co-precipitated in NaOH solution to make MNPs. The MNPs were then coated with 10% TEOS (Tetraethyl orthosilicate), functionalized via 16-PHDA, and immobilized with α-transglucosidase with high binding efficiency (82%)^24^.

### Characterization

#### FT-IR analysis

**Figure 4:**
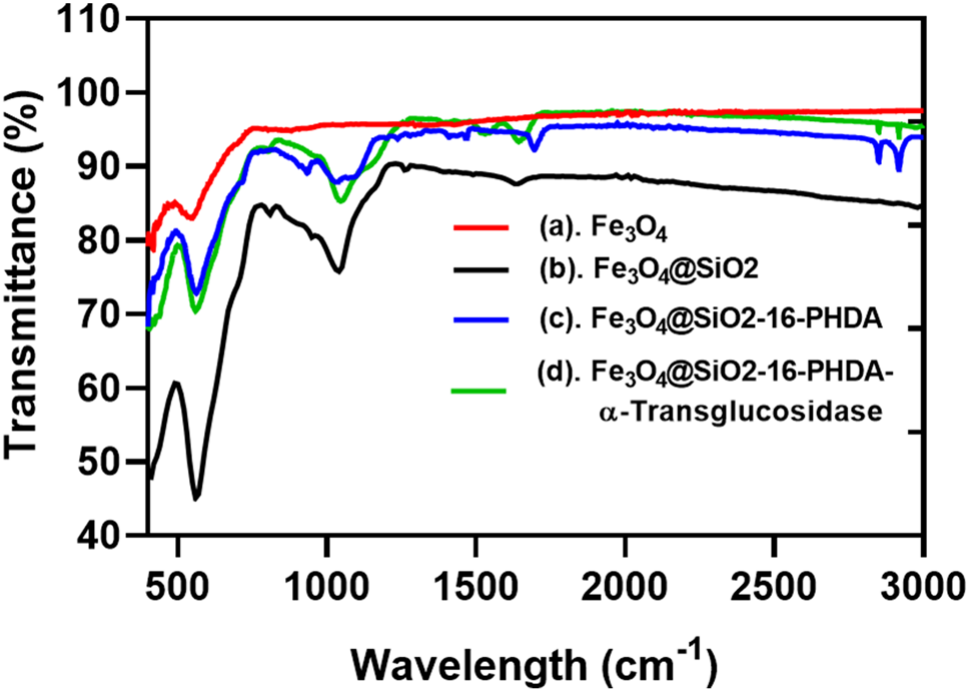
FTIR spectra of α-transglucosidase immobilized on MNPs. (**a**). Fe_3_O_4;_ (**b**). Fe_3_O_4_@SiO_2;_ (c). Fe_3_O_4_@SiO_2_-16-PHDA; (d). Fe_3_O_4_@SiO_2_-16-PHDA-α-transglucosidase.

FT-IR spectroscopy analysis data (Fig. 4) confirmed the formation of MNPs and the surface modifications. The functional group of FeT–O–FeO was observed around 600–550 cm^−1^ in MNPs. Due to the Si–O–Si bond, a stretching peak displayed around 1031.01–1080 cm^−1^, confirming the magnetite nanoparticles silica coating. The stretching peak was around 1100–900 cm^−1^ confirming P–O and P–O–Fe groups after 16-PHDA linking. The spectral signal at 1643.32 cm^−1^ confirmed the presence of the amide carbonyl (NH–CO) group of immobilized α-transglucosidase during immobilization. The FTIR results also agreed with the previously reported study^24^.

#### TEM analysis

**Figure 5:**
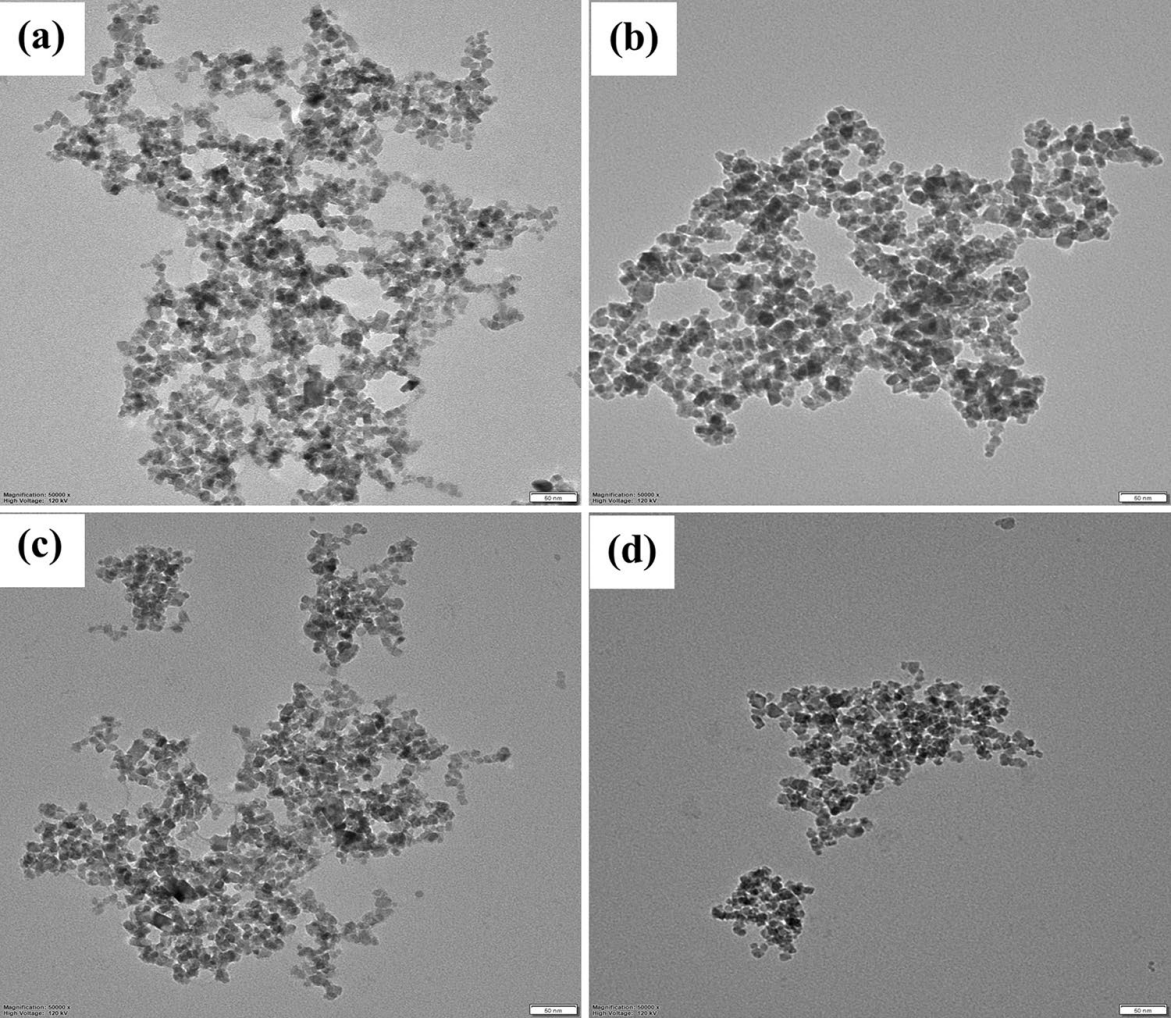
TEM image of (**a**). Fe_3_O_4_, (**b**). Fe_3_O_4_@SiO_2_ (**c**). Fe_3_O_4_@SiO_2_-16-PHDA; (**d**). Fe_3_O_4_@SiO_2_-16-PHDA-α-transglucosidase (Scale bar 50 nm).

The size distribution and TEM images of the Fe_3_O_4_, Fe_3_O_4_@SiO_2_, Fe_3_O_4_@SiO_2_-16 PHDA, and Fe_3_O_4_@SiO_2_-16 PHDA-α-transglucosidase all support the quasi-spherical shape of the nanoparticles as showed in Fig. 5. The predicted diameter of uncoated Fe_3_O_4_ nanoparticles is around 20 nm which further increases to 35 nm following silica coating, as illustrated in Fig. 5. According to TEM data, nanoparticles that have been modified with a linker and an enzyme have little to no change in diameter, which also in agreement with previous study^26^. This could be because of their smaller electron density as organic molecules only provide inadequate TEM disparity.

#### FESEM analysis

**Figure 6:**
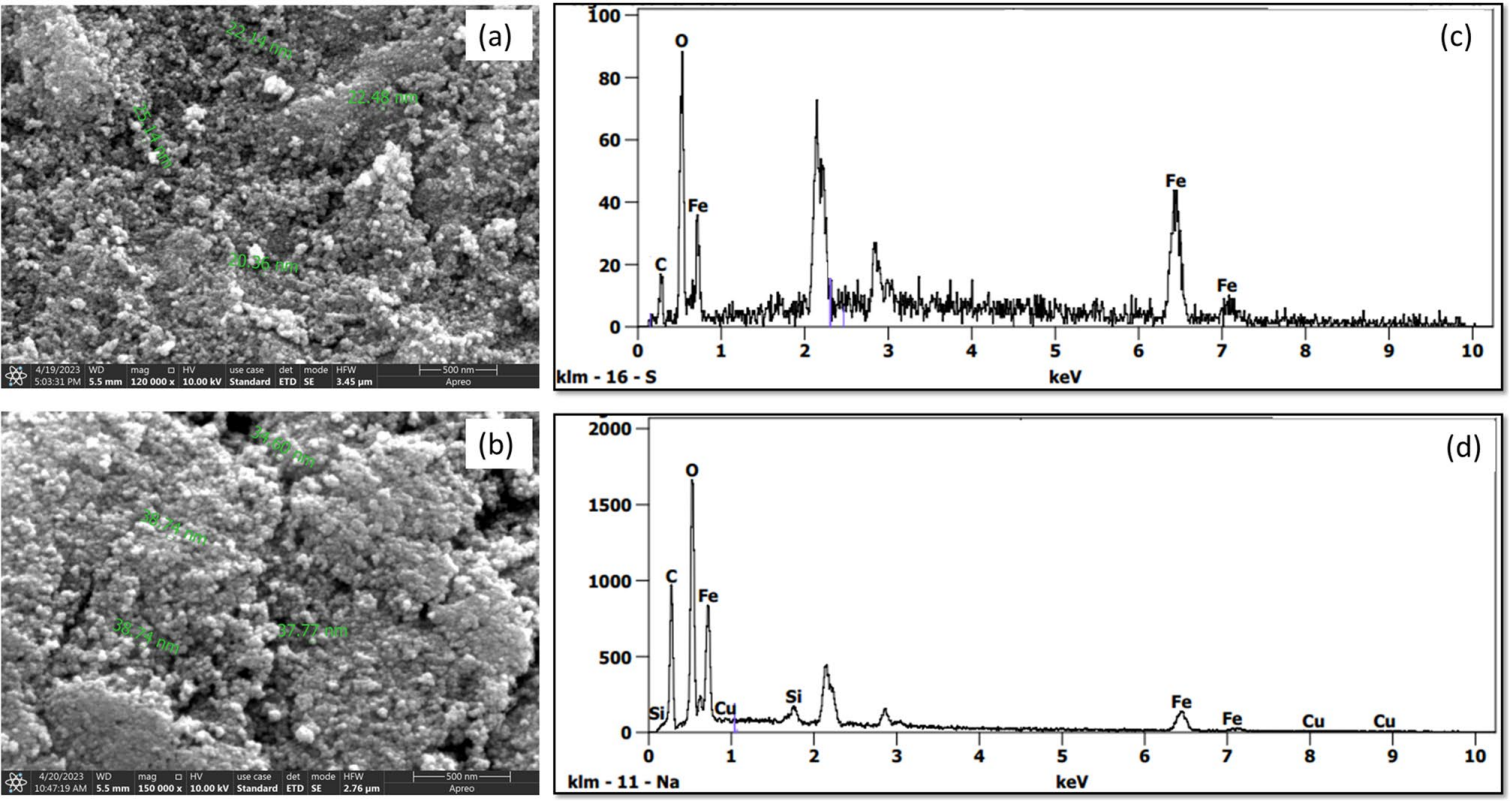
FESEM image of (**a**); Fe_3_O_4_ (**b**). Fe_3_O_4_@SiO_2_; EDX image of (**c**). Fe_3_O_4_ and (**d**). Fe_3_O_4_@SiO_2._

Field emission scanning electron microscopy investigated surface morphology and particle size/shape. The results showed in Fig. 6a, the average size of the sphere-shaped Fe_3_O_4_ particles was 20–25 nm. Further, Fe_3_O_4_ particles sphere size slightly increased about 34–38 nm after silica coating on the surface of the Fe_3_O_4_ particles (Fig. 6b), comparable with previous study^26,46,47^. There was no significant change in the physical appearance of the nanoparticles occurred following modification with 16-PHDA and α-trasglucosidase enzyme.

#### EDX analysis

The energy dispersive X-ray (EDX) spectroscopy results were used to characterize the mass percent composition of the synthesized Fe_3_O_4_ and Fe_3_O_4_ /SiO_2_ particles. Figure 6c showed Fe_3_O_4_ had a mass percent composition of 64.8% Fe, 31.4% O, and 3.9% C. After silica coating, the atomic composition of the nanospheres were Fe 63.0%, O 23.6%, C 8.9%, and Si 3.7% as depicted in Fig. 6d. The overall results were in agreement with previous study^46^.

#### XRD analysis

**Figure 7:**
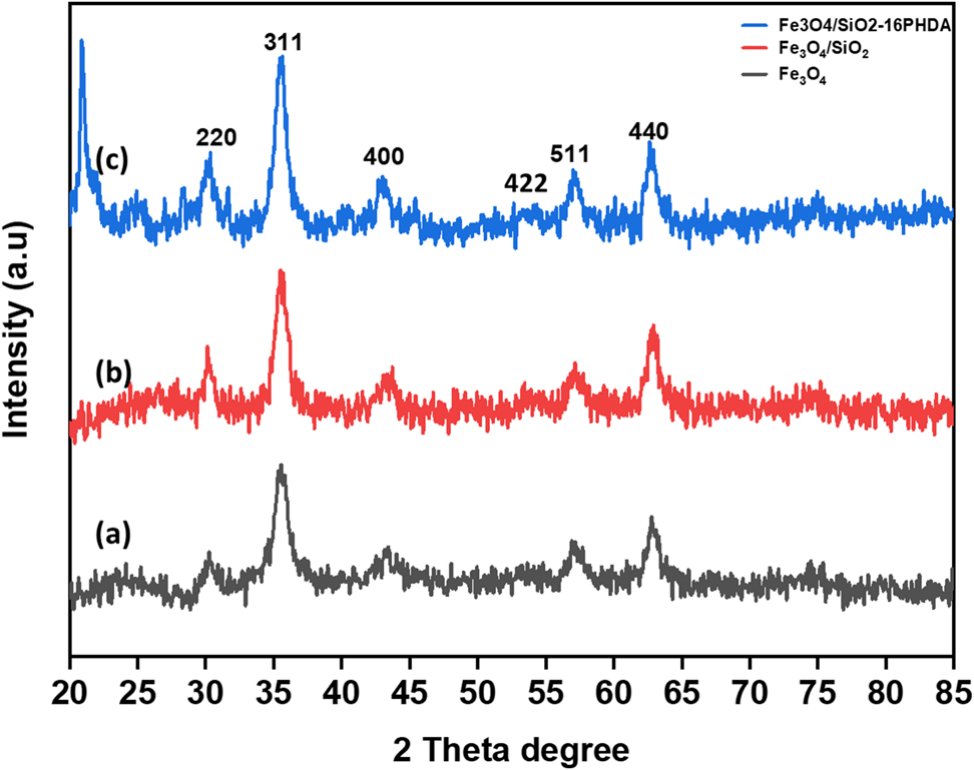
XRD spectra of (**a**). Fe_3_O_4_ (**b**). Fe_3_O_4_@SiO_2_ (**c**). Fe_3_O_4_@SiO_2_-16-PHDA.

X-Ray Diffraction Spectroscopy (XRD) analysis was performed to confirm the purity and crystallinity of Fe_3_O_4_, Fe_3_O_4_@SiO_2_, organophosphorus linker, and enzyme-modified nanoparticles. Figure 7 showed the magnetite nanoparticles diffractogram peaks at 2θ = 30.2°, 35.6°, 43.2°, 57.3° and 62.9° attributed to (220), (311), (400), (422), (511) and (440) reflections, demonstrating the acceptable crystallinity of nanoparticles which was comparable with previously published data^26,36^. The overall result suggested that MNPs did not change crystalline structure after enzyme immobilization.

#### TGA analysis

**Figure 8:**
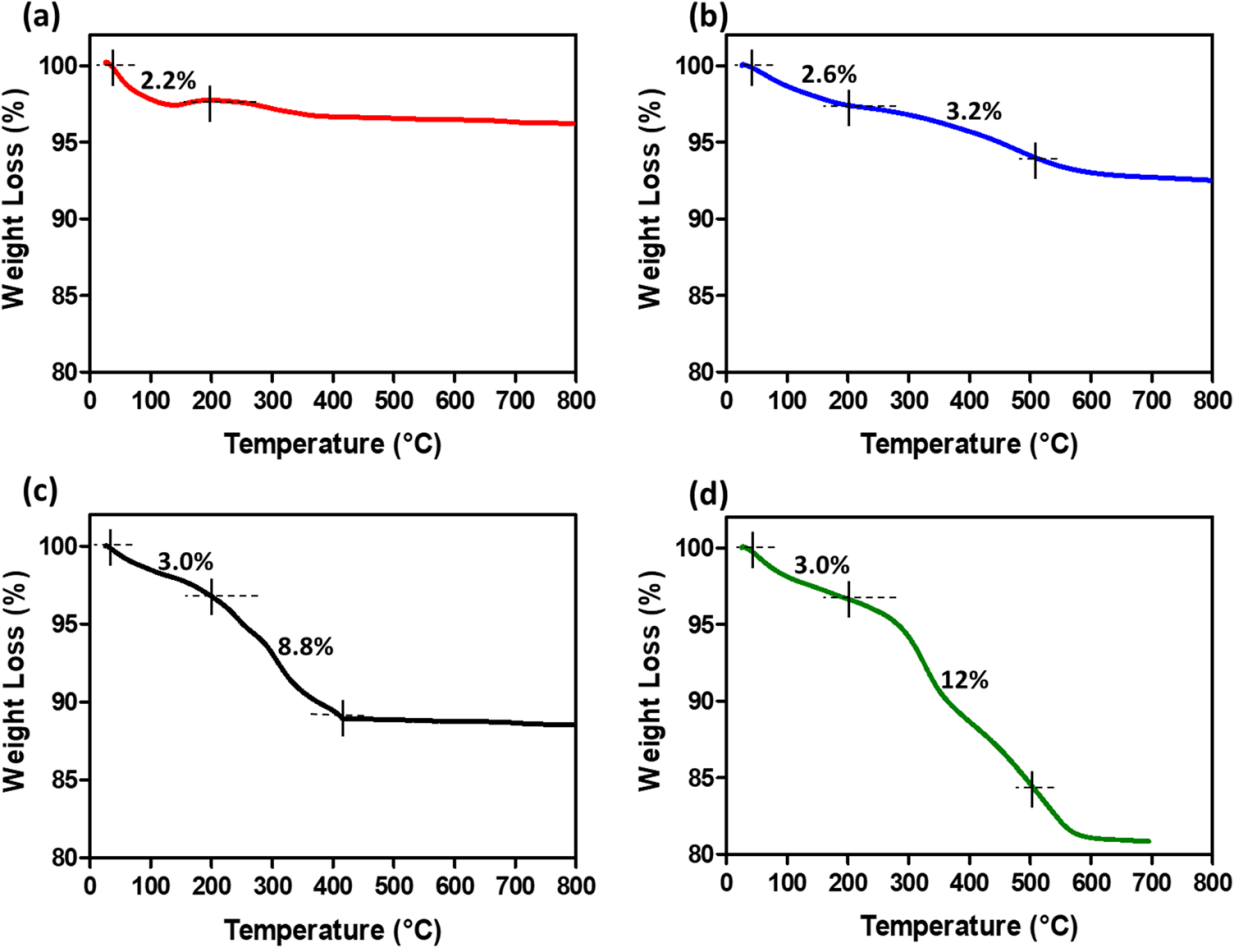
Thermogravimetric analysis (TGA) of (**a**). Fe_3_O_4_ (**b**). Fe_3_O_4_@SiO_2_ (**c**). Fe_3_O_4_@SiO_2_-16-PHDA (d). α-transglucosidase bound Fe_3_O_4_@SiO_2_-16-PHDA.

TGA analysis was performed to determine the percentage weight loss of Fe_3_O_4_, Fe_3_O_4_@SiO_2_, Fe_3_O_4_@SiO_2_-16 PHDA, and Fe_3_O_4_@SiO_2_-16 PHDA-α-transglucosidase (Fig. 8). The samples were heated to a range of 25–800 °C to analyse the percentage loss of weight. At temperatures below 200 °C, all samples exhibited a minor initial mass loss due to the desorption of adsorbed water. As the temperature increased above 200–500 °C, weight loss was not observed in Fe_3_O_4_ nanoparticles. However, weight losses of about 3.2%, 8.8.%, and 12.0% were recorded in Fe_3_O_4_@SiO_2_, Fe_3_O_4_@SiO_2_-16-PHDA, and Fe_3_O_4_@SiO_2_-16-PHDA-α-transglucosidase respectively which were also in accordance to previously published data^36^. The Additional loss of around 3.2% confirmed the successful binding of α-transglucosidase on MNPs.

#### DLS analysis

Zeta potential was used to assess how the surface charge of the enzyme and MNPs in the solution affected the stability of its colloidal particles. The stability of the colloidal solution would increase with increasing zeta potential. The results of measuring the zeta potential of Fe_3_O_4,_ Fe_3_O_4_@SiO_2_, Fe_3_O_4_@SiO_2_-16-PHDA, and Fe_3_O_4_@SiO_2_-16-PHDA-α-transglucosidase are showed in Table 2. The zeta potential of Fe_3_O_4_@SiO_2_-16-PHDA increased to positive levels (from −46.0 to −31.0) after α-transglucosidase immobilization, indicating that less negative α-transglucosidase neutralized the negatively charged MNPs which also in agreement with the previous study^24^. Further the PDI values of all the MNPs in the range of 0.161–0.212 suggested higher colloidal stability.

**Table 2:** Summary of polydispersity index (PDI), and zeta potential parameters of Fe_3_O_4_, Fe_3_O_4_@SiO_2_, Fe_3_O_4_@SiO_2_-16PHDA, Fe_3_O_4_@SiO_2_-16 PHDA-α-transglucosidase.

Samples	PDI	ζ-P (Mv)
Fe_3_O_4_	0.212 ± 0.01	− 37.0 ± 1.52
Fe_3_O_4_@SiO_2_	0.22 ± 0.010	− 43.0 ± 0.57
Fe_3_O_4_@SiO_2_-16 PHDA	0.208 ± 0.04	− 46.0 ± 1.0
Fe_3_O_4_@SiO_2_-16PHDA-α-transglucosidase	0.161 ± 0.01	− 31.0 ± 1.15

### Stability and Reusability of immobilized α-transglucosidase

**Figure 9:**
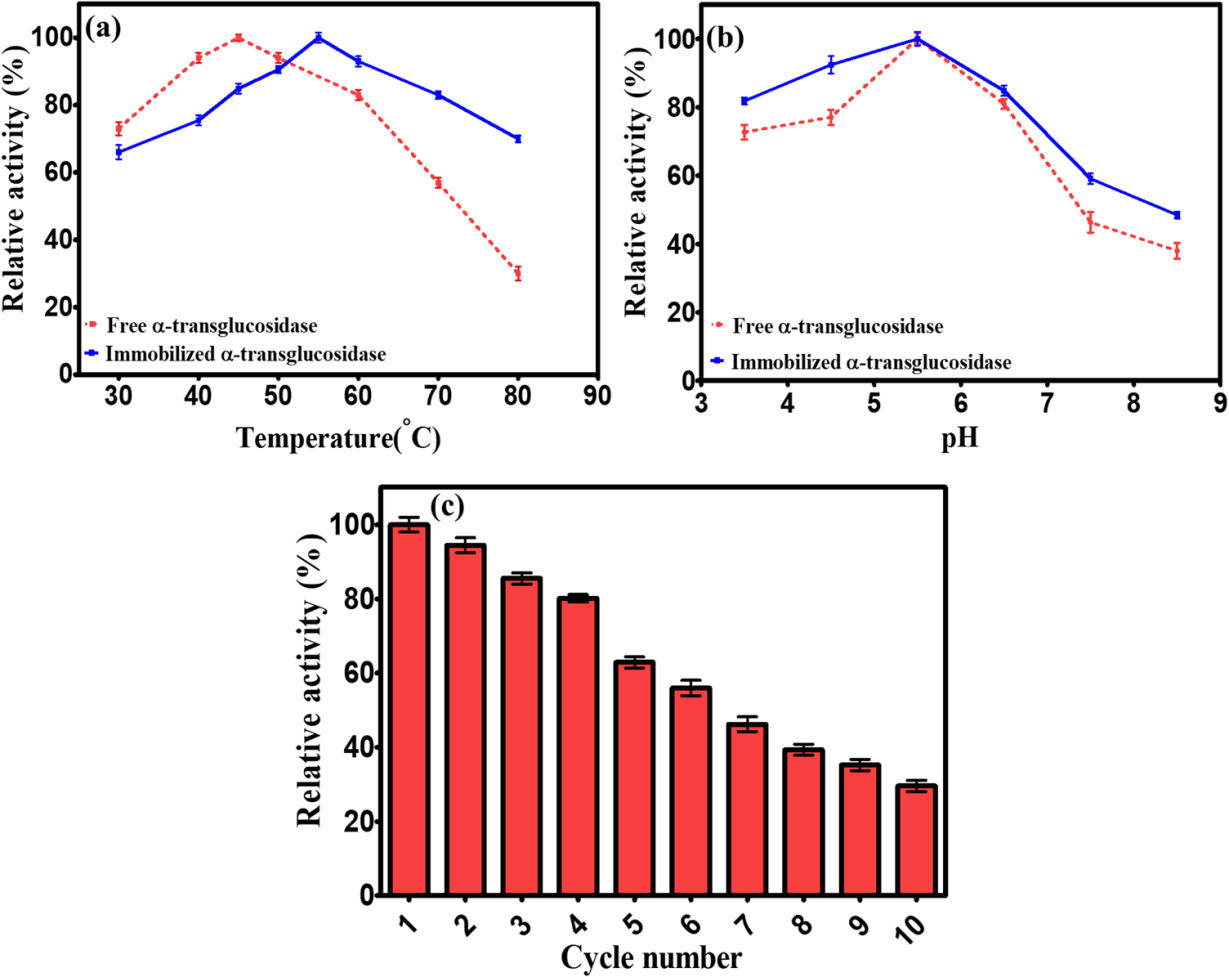
Stability of free and immobilized α-transglucosidase (**a**). at different temperature range 30–80 °C and (**b**). pH range 3.5–8.5. (**c**). Reusability of immobilized α-transglucosidase in subsequent 10 cycles of reaction conducted at optimized conditions (6.9 U g^-1^, 45 °C, 9 h and pH 5.5). The first cycle enzyme activity considered as 100%. All data are represented as mean ± SD (n = 3).

Free and immobilized α-transglucosidase activities were measured in the temperature and pH range of 30–80 °C and 3.5–8.5, respectively. The results revealed that the optimum temperature for free α-transglucosidase was 45 °C which was slightly pushed towards 55 °C for immobilized α-transglucosidase (Fig. 9a). Further, significant reduction (30% at 80 °C) in the activity of the free α-transglucosidase whereas the immobilized enzyme retained nearly 70% activity at 80 °C. Figure 9b revealed that free and immobilized α-transglucosidase exhibited nearly similar activity at optimum pH 5.5. However, higher retention in the activity was observed for immobilized α-transglucosidase compared to the free enzyme at pH 8.5. The overall result was also in agreement with previous studies suggesting that magnetic nanoparticle immobilization improved the thermal and pH stability^37^.

The advantage of MNPs is easy removal from a reaction mixture by using simple magnet which provide magnetic nanoparticle stand-alone-candidate to other nonmagnetic nanoparticles. The result showed in Fig. 9c clearly demonstrated that immobilized α-transglucosidase retained its activity up to more than 60% after 5 reaction cycles. Further, it was observed that the maximum yield of IMOs (64 g l^-1^ ± 2.3) was achieved with immobilized α-transglucosidase at the optimum condition as enzyme to substrate ratio 6.9 U g^-1^, reaction time 9 h, reaction temperature 45 °C, and pH 5.5^24^.

### Analysis of enzyme kinetics parameter of α-transglucosidase

The analysis of enzyme kinetic parameters of free and immobilized α-transglucosidase such as, Michaelis–Menten constant (K_m_), turnover number (K_cat_), and catalytic efficiency (K_cat_/K_m_), was evaluated using a GraphPad Prism software version 5^43^. The enzymatic parameters were calculated using Michaelis–Menten and Lineweaver–Burk plots. Fig. S4 illustrates the outcomes of the α-transglucosidase kinetic study at different maltose concentrations. The result showed up to 300 mM maltose, the reaction rate and maltose concentration are correlated linearly. The reaction rate was stabilized when the concentration of maltose was larger than 300 mM. The results showed in Table 3 support the findings in Fig. S4 in their observations. The K_m_ values of free and immobilized enzymes were 97.85 ± 11.40 and 81.08 ± 9.990, respectively. The best-fit value of K_cat_ is 1107 ± 14.28 and 715.6 ± 9.528 for free and immobilized enzymes^39^.

**Table 3:** Kinetic parameters (K_m,_ K_cat_ and K_cat_/ K_m_) of α-transglucosidase and MNPs@ α-transglucosidase for hydrolysis of maltose as a substrate.

Kinetic parameter	Free enzyme (α-transglucosidase)	MNPs@α-transglucosidase
K_m_ (mM)	97.85 ± 11.40	81.08 ± 9.990
K_cat_ (s^-1^)	1107 ± 14.28	715.6 ± 9.528
K_cat_/K_m_ (mM^-1^ s^-1^)	11.3	8.82
R^2^	0.98	0.97

### Thin layer chromatography and HPAEC-PAD analysis

TLC and HPLC were used to analyse the transglucosylation reaction products (Fig. S5). As the hydrolysis time increased from Lane 1 (6 h) to Lane 5 (30 h), glucose concentration increased, and oligosaccharide concentration decreased, as showed in Fig. S5a. This is due to the fact that oligosaccharides degrade into glucose as time goes on, making the glucose band darker over time and the oligosaccharide band disappear. Fig. S5b showed HPAEC-PAD chromatogram of transglucosylation reaction products after 6 h hydrolysis time^18^.

### Size-exclusion chromatography purification and MALDI-TOF–MS analysis of IMOs

The product obtained after transglucosylation of saccharified starch was separated by size-exclusion chromatography. Further, HPAEC-PAD analysis revealed the glucose content in the purified IMOs rich fraction was reduced to 4 g l^-1^ (Supplementary data Fig. S6). The other constituents in the purified fractions were to be as; isomaltose: 37.7 g l^-1^, isomaltotriose: 21.6 g l^-1^, panose: 17 g l^-1^, maltose: 3 g l^-1^, and another oligosaccharide: 20 g l^-1^. The purity of the IMOs rich fraction was nearly 85% which was nearly similar as described in the previous study^48^.

**Figure 10:**
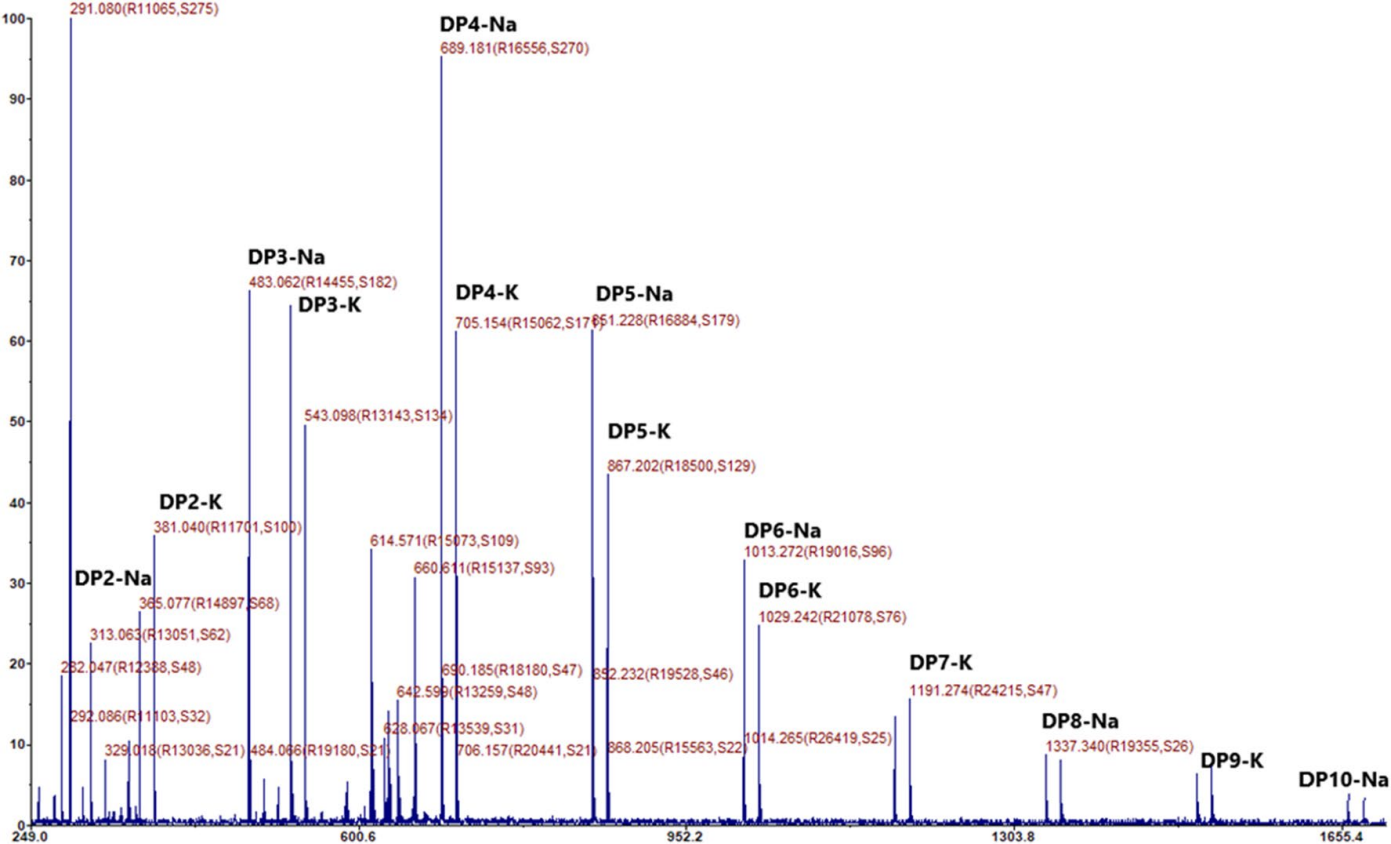
MALDI-TOF–MS spectroscopy of the formed IMOs generated after the treatment of saccharified starch with α-transglucosidase.

MALDI-TOF–MS was used to analyze the purified IMOs. The analysis showed the presence of oligosaccharides DP 2–10 (m/z 365–1337 Da) showed in Fig. 10, comparable to previously published data^49^. However, according to m/z peak analysis, the amount of oligosaccharides DP > 10 was very low and undetectable.

#### Methylation analysis of IMOs oligosaccharides

The glycosyl linkage analysis of IMOs indicates that terminal glucopyranosyl (Glcp), α-(1 → 4), and α-(1 → 6) D linked Glcp residues were more abundant in the molar ratio of nearly 1.02:0.43:1.0 with the small amount of α-(1 → 2) and α-(1 → 3)—linked Glcp residues in the relative molar ratio of nearly 0.12 and 0.05. The linkage analysis data is shown in Table 4, and detailed figures are provided in the supplementary data (Supplementary Fig. S7). The study further indicated that IMOs contain α-(1 → 4) and α-(1 → 6)- linked Glcp residues as the primary linkage present in isomaltose, isomaltotriose, and panose which was comparable with previously reported study^50^.

**Table 4:** Linkage analysis of PMAA derivative of IMOs, partially methylated alditol acetate in the current study.

PMAA derivative	Deduced linkage	Molar^a^ ratio	Mass fragment
2,3,4,6-tetra-O-methyl-D-glucitol	Terminal-D-glucopyranosyl	1.02	205, 161, 118, 162
3,4,6-tri-O-methyl-D-glucitol	α-D-Glcp-(1 → 2)- α-D-Glcp	0.12	161, 190
2,4,6-tri-O-methyl-D-glucitol	α-D-Glcp-(1 → 3)- α-D-Glcp	0.05	277, 161, 118, 234
2,3,6-tri-O-methyl-D-glucitol	α-D-Glcp-(1 → 4)- α-D-Glcp	0.43	233, 118
2,3,4-tri-O-methyl-D-glucitol	α-D-Glcp-(1 → 6)- α-D-Glcp	1.0	233, 189, 118, 162

^a^Molar ratio relative to the 6-Glc, 4-Glc, 3-Glc and 2-Glc residues.

#### NMR analysis

The purified IMOs in which fraction was characterized by ^1^H, ^13^C, and 2D NMR (TOCSY, and HSQC) NMR spectroscopy (supplementary Fig. S8).

^1^H NMR spectroscopy showed the anomeric signal at δ 5.225(α) and δ 4.649 (β) in arrangement with a reducing α-(1 → 4) D Glcp unit. Further, the groups of anomeric signals along δ 5.389–5.395 and 4.970 revealed the presence of α-(1 → 4) and α-(1 → 6) linkage, respectively. The α-(1 → 6): α-(1 → 4) linkage ratio was found to be about 1.0:0.67, which suggested the presence of significantly higher α-(1 → 6) linkages. The result was also in agreement with the ratio of α-(1 → 6): α-(1 → 4) linkage obtained by glycosyl linkage analysis.

^13^C chemical shift was deduced from ^13^C and HSQC measurements. The analysis revealed the presence of anomeric signals of α-(1 → 4) and α-(1 → 6) linkage in the range of δc 100.7 and 98.3, respectively. The anomeric signals of δc 92.6 and 96.6 were indicative of reducing α-(1 → 4) D-Glcp and β-(1 → 4) residues, respectively. The anomeric signals agreed with previously reported results^11^. The anomeric signal of δ_H_/δ_C_ at 5.427/90.2 and 4.785/97.1 suggested the presence of reducing α-(1 → 2) D-Glcp and β-(1 → 2) D-Glcp residues. Furthermore, the anomeric signal at δ_H_/δ_C_ at 5.233/93.2 and 4.663/96.8 were indicative of reducing α-(1 → 3) D-Glcp and β-(1 → 3) residues. The detailed NMR analysis is summarized in Table 5.

**Table 5:** ^1^H/^13^C chemical shifts (ppm, D2O, 300 K) of Isomaltooligosaccharide obtained by the incubation of maltooligomers with α-transglucosidase.

Residue	H1/C1	H2/C2	H3/C3	H4/C4	H5/C5	H6a/C6	H6b/C6
-(1 → 4)-α-D-Glcp-(1 → 4)-	5.395/100.7	3.621/72.0	3.963/74.3	3.664/77.8	3.841/72.2	3.882/61.5	3.812/61.5
α-D-Glcp-(1 → 4)-	5.404/100.4	3.574/72.5	3.681/73.5	3.411/70.0	3.718/73.3	3.849/60.9	3.761/60.9
Rα, -(1 → 4)-D-Glcpα	5.225/92.6	3.561/72.4	3.976/74.0	3.642/77.7	3.943/70.8	3.849/61.4;	3.796/61.4
Rβ, -(1 → 4)-D-Glcpβ	4.649/96.6	3.272/75.0	3.761/77.0	3.622/77.7	3.599/75.3	3.903/61.5	3.751/61.5
-(1 → 6)-α-D-Glcp-(1 → 6)-	4.970/98.3	3.578/72.3	3.725/73.8	3.499/70.4	3.931/70.8	3.782/66.5	3.983/66.5
α-D-Glcp-(1 → 6)-	4.984/98.3	3.556/72.4	3.751/73.9	3.422/70.5	3.751/72.5	3.851/61.2	3.781/61.2
Rα, -(1 → 6)-D-Glcpα	5.239/93.0	3.551/72.4	3.718/73.8	3.531/70.3	4.015/70.9	3.705/66.7	3.999/66.7
Rβ, -(1 → 6)-D-Glcpβ	4.672/97.1	3.252/75.0	3.448/76.9	3.521/70.3	3.642/75.2	3.781/66.7	3.961/66.7
-(1 → 6)-α-D-Glcp-(1 → 4)	5.389/100.7	3.604/72.5	3.69/73.8	3.507/70.1	3.91/72.1	3.726/66.8	3.975/66.8
α-D-Glcp-(1 → 2)-	5.084/97.6	3.551/72.3	3.781/73.8	3.452/70.5	3.935/72.9	3.842/61.0	3.771/61.0
Rα, -(1 → 2)-D-Glcpα	5.427/90.2	3.632/77,1	3.807/72.2	3.452/70.7	3.852/72.2	3.842/61.0	3.761/61.0
Rβ, -(1 → 2)-D-Glcpβ	4.785/97.1	3.373/79.9	3.551/75.4	3.437/70.9	3.452/76.5	3.867/61.5	3.688/61.0
α-D-Glcp-(1 → 3)-	5.371/99.8	3.571/72.5	3.751/73.8	3.437/70.3	4.015/72.5	3.842/61.2	3.781/61.2
Rα, -(1 → 3)-D-Glcpα	5.233/93.2	3.633/70.8	3.852/nd	3.650/70.8	3.842/72.0	3.876/61.0	3.751/61.0
Rβ, -(1 → 3)-D-Glcpβ	4.663/96.8	3.331/73.6	3.642/83.2	3.642/70.8	3.474/76.5	3.878/61.5	3.728/61.5

## Conclusions

IMOs have been produced by potato peel starch through three-step liquefaction, saccharification, and transglucosylation. A recombinant α-transglucosidase from *A. Niger* has been produced in *E. coli*. The α-transglucosidase was immobilized with MNPs for reusability (5 cycles more than 60% activity) and characterized by using FT-IR, TEM, FESEM, EDX, XRD, TGA, and DLS analysis. The maximum IMOs yield (70 g l^-1^) was achieved by RSM. The detailed structural characterization of IMOs suggested DP in ranges of 2–10 with the presence of α- (1 → 4) and α- (1 → 6) -D-Glcp residues as major constituents.

### Supplementary Information

Supplementary Information.

## Data Availability

The datasets used and/or analysed during the current study available from the corresponding author on reasonable request.
