# Supplementary material for: Immobilization of α-transglucosidase on silica-coated magnetic nanoparticles and its application for production of isomaltooligosaccharide from the potato peel
Source: Sci Rep. 2023 Aug 5;13:12708. doi: 10.1038/s41598-023-38266-8 (PMC10404235; doi:10.1038/s41598-023-38266-8)
Supplement: Supplementary file 1 — Supplementary Information. [file 41598_2023_38266_MOESM1_ESM.docx]

**Immobilization of α-transglucosidase on silica-coated magnetic nanoparticles and its application for production of isomaltooligosaccharide from the potato peel**

**Rohit Maurya^1,2^, Usman Ali^1^, Sunaina Kaul^1^, Raja Bhaiyya^3^, Ravindra Pal Singh^1,3^, Koushik Mazumder^1^***

**^1^**National Agri-Food Biotechnology Institute (NABI), Sector-81 (knowledge City), S.A.S. Nagar, Mohali-140306, Punjab, India.

^2^Regional Centre for Biotechnology, Faridabad-Gurgaon Haryana 121001

^3^Gujarat Biotechnology University, Near Gujarat International Finance Tech-City, Gandhinagar, Gujarat 382355.

*Corresponding author: Tel: +91-172-5221244, Fax: +91-1725221100, E-mail address: koushik@nabi.res.in

**Supplementary data**

### *Effect of different parameters on saccharification*

### Fungamyl^®^ was used for the saccharification reaction. Fungamyl^®^ enzyme converts large oligosaccharides into maltooligosaccharides. The more noticeable maltooligosaccharides contents were maltose (72 g.l^-1^) and maltotriose (18 g.l^-1^) with a minor amount of glucose (13 g.l^-1^). The Fungamyl^®^ from *Aspergillus oryzae* exhibited their maximum activity at optimum condition: dose of Fungamyl^®^ was 1.2 U.g^-1^ (Fig. S1 A) for liquefied starch at 4 h (Fig. S1 B), pH 6.5 (Fig.S1 C), and temperature 50°C (Fig. S1 D).

###
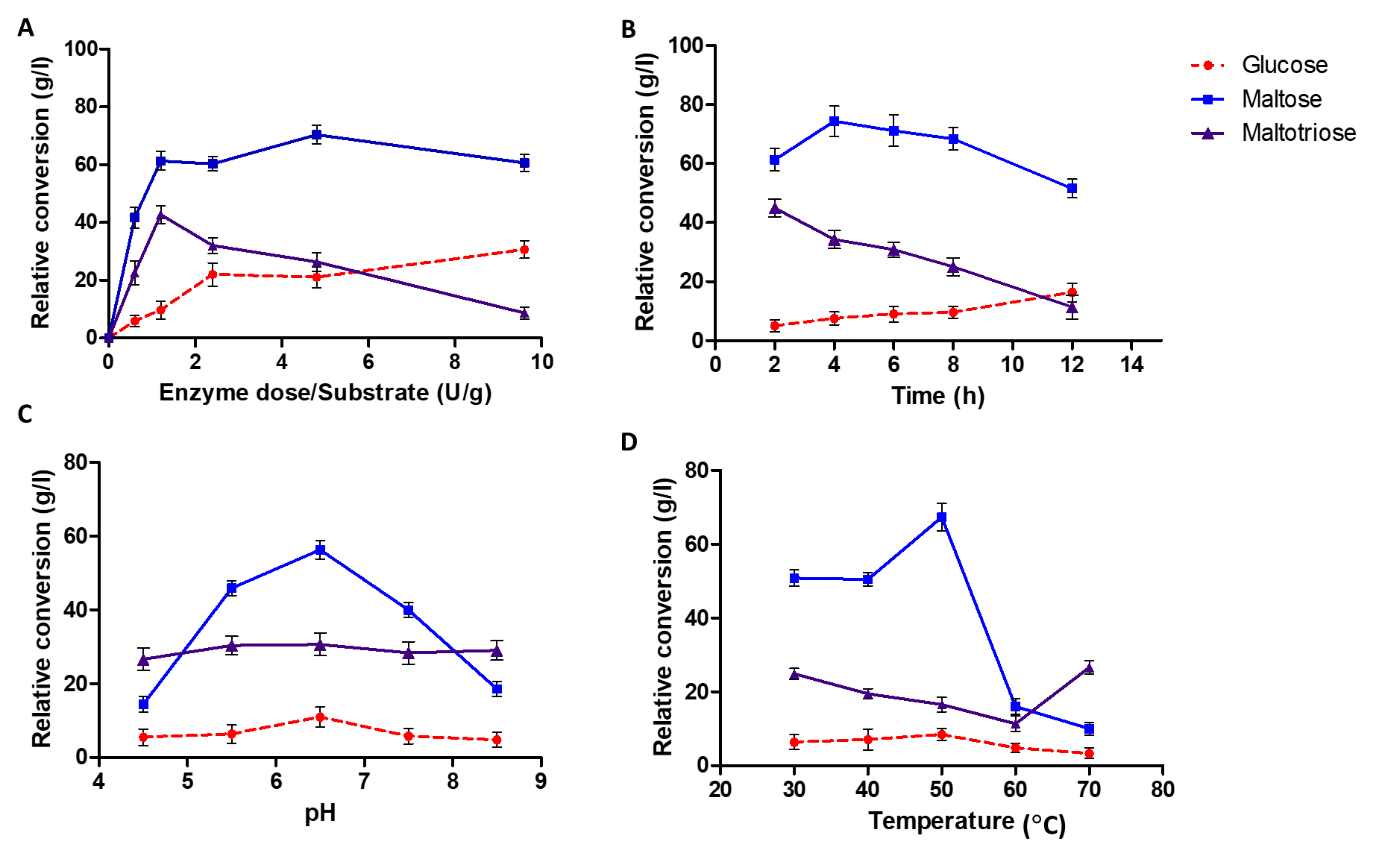


### Figure. S1. Effect of different parameters on saccharification reaction A. Enzyme dose substrate ratio; B. Reaction time (h); C. Reaction mixture pH; D. Temperature (°C).

###
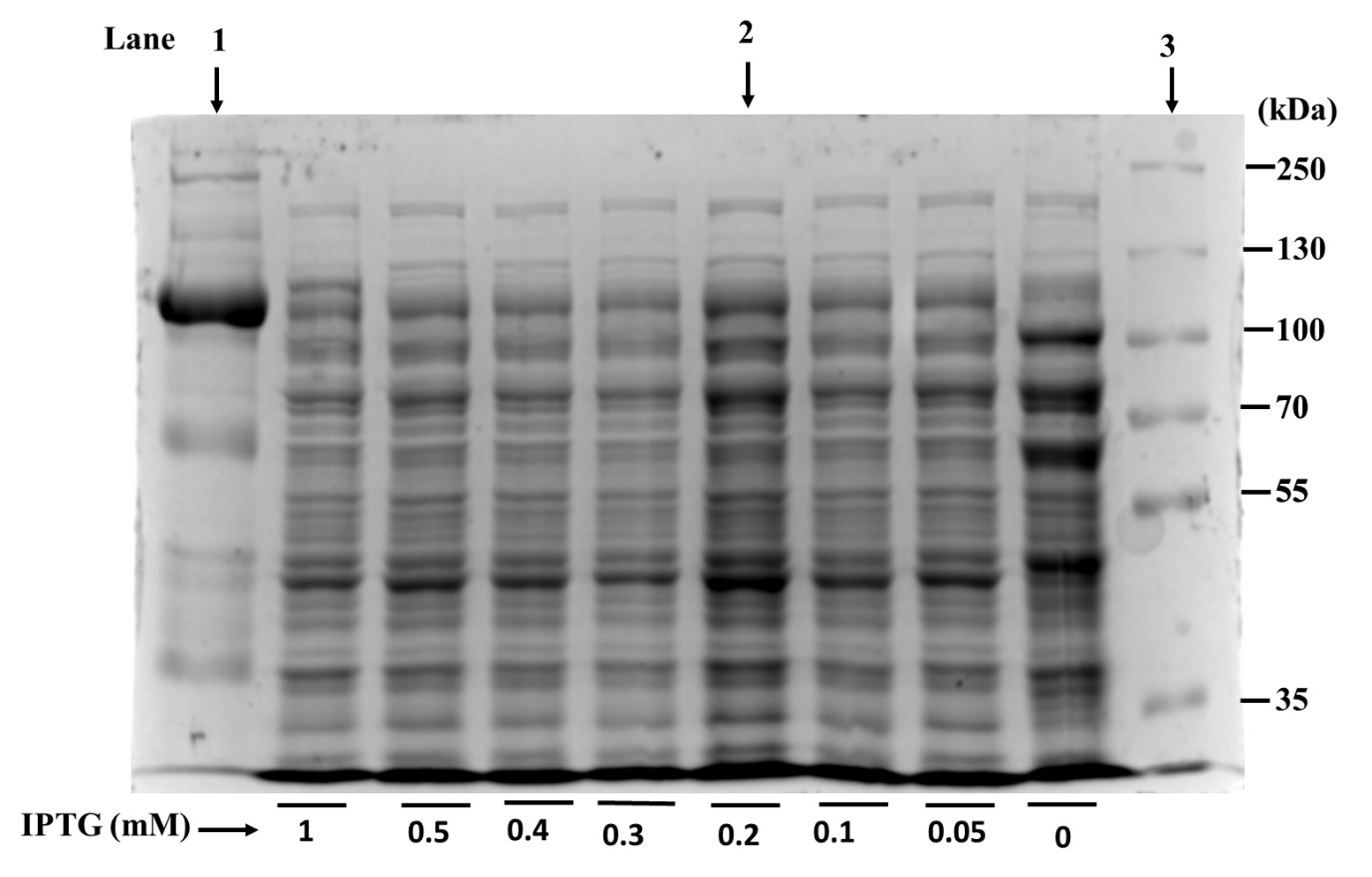


### Figure. S2. Original image of SDS PAGE showed Lane 1 purified α-transglucosidase; lane 2 Crude enzyme (0.2 Mm IPTG); lane 3 protein marker which are presented in manuscript Figure. S1. Other lane represented optimization of protein overexpression at different IPTG concentration (0-1 mM).

### *Effect of different parameters on transglucosylation*

### The last step of transglucosylation was the most important step because in this step α-(1-4) glycosidic linkage converted into α-(1-6) glycosidic linkage. Enzyme to substrate ratio, time, temperature, and pH were important factors in this process. As shown in Fig. S3 A, the yield of IMOs was increased with the elevation of enzyme to substrate ratio from 0.7 U.g^-1^ to 5.6 U.g^-1^. The yield of IMOs increased to 55.8 g.l^-1^. Furthermore, the addition of enzymes more than 5.6 U.g^-1^ led to a decrease in the yield of IMOs and increased in the glucose concentration. Therefore, the optimal enzyme to substrate ratio was determined to be 5.6 U.g^-1^. The pH was the second important factor in the enzymatic hydrolysis process because enzyme activity is greatly affected by the pH. As showed in Fig. S3C, the yield of IMOs was increased with the increasing pH from 3.5 to 5.5; the largest yield of IMOs was 58 g.l^-1^ at pH 5.5. Above pH 5.5, and below 4.5 the yield was decreased, which may be attributed to the inhibition of the α-transglucosidase activity. Another factor that affects IMOs yield was reaction time. In Fig. S3B, showed the IMOs yield increased but maltose and maltotriose were decreased with increasing reaction time from 1h to 6 h. Furthermore, the yield of IMOs was decreased and the glucose concentration increased from reaction time above 6h. The temperature was the last factor as shown in Fig. S3D as temperature increased from 35 to 45°C the IMOs yield reached a maximum of 58 g.l^-1^ at 45°C, after that the yield of IMOs decreased significantly with increased glucose level. Therefore, the optimal temperature was determined to be 45°C. The previous study also demonstrated that the IMOs ( isomaltose, isomaltotriose, panose) increased slowly at first and then decreased with increasing glucose content.

###
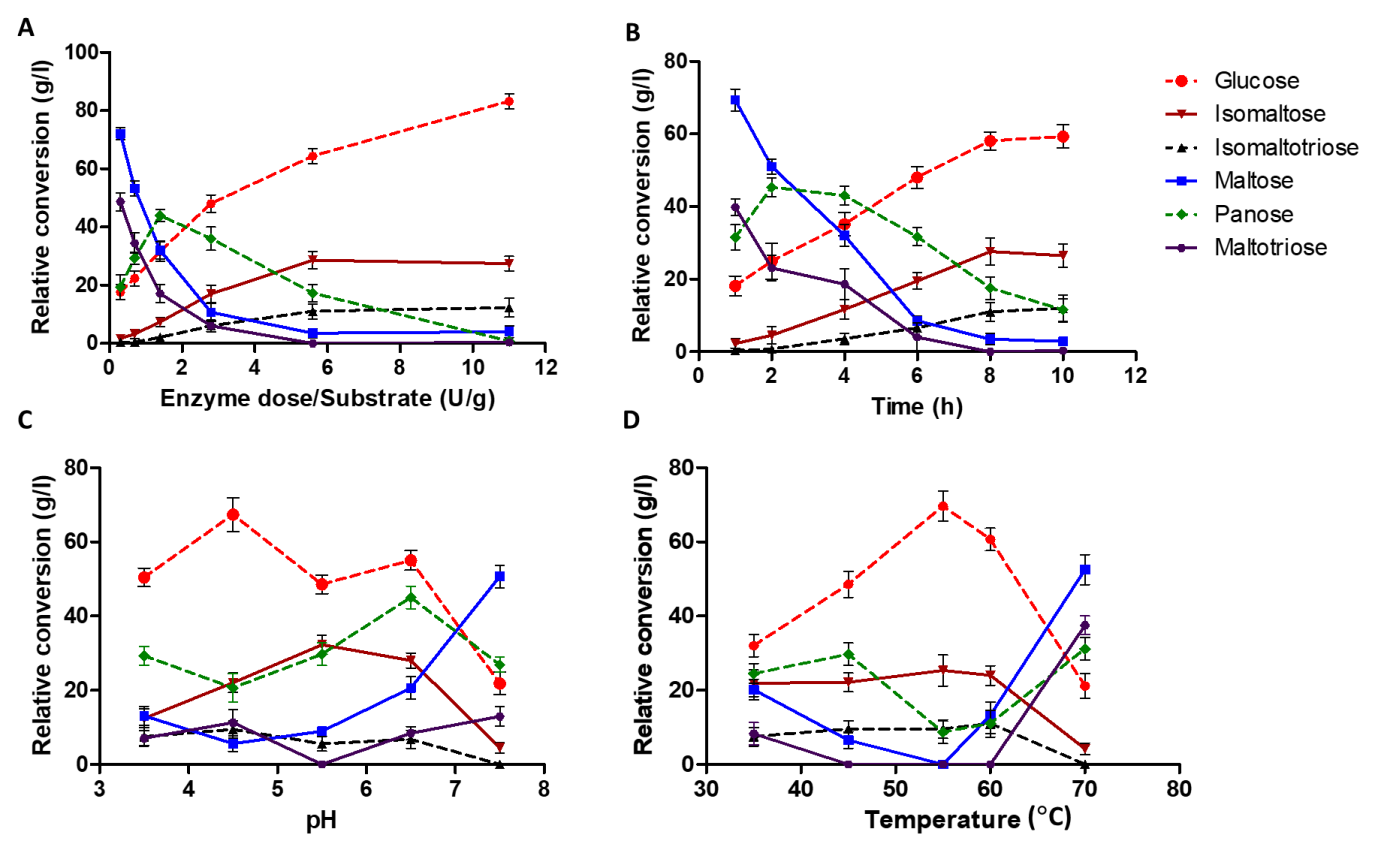
 Figure. S3. Effect of different parameters on transglucosylation reaction; A. Enzyme dose substrate ratio; B. Reaction time (h); C. Reaction mixture pH; D. Temperature (°C).

###
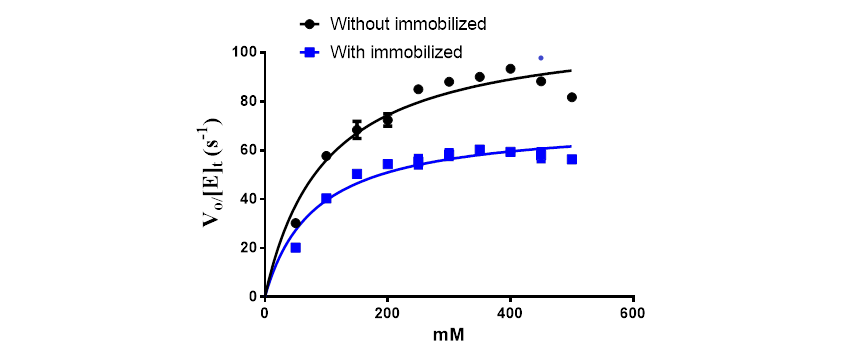


### Figure. S4. Enzyme kinetics parameter with and without immobilization of α- transglucosidase.


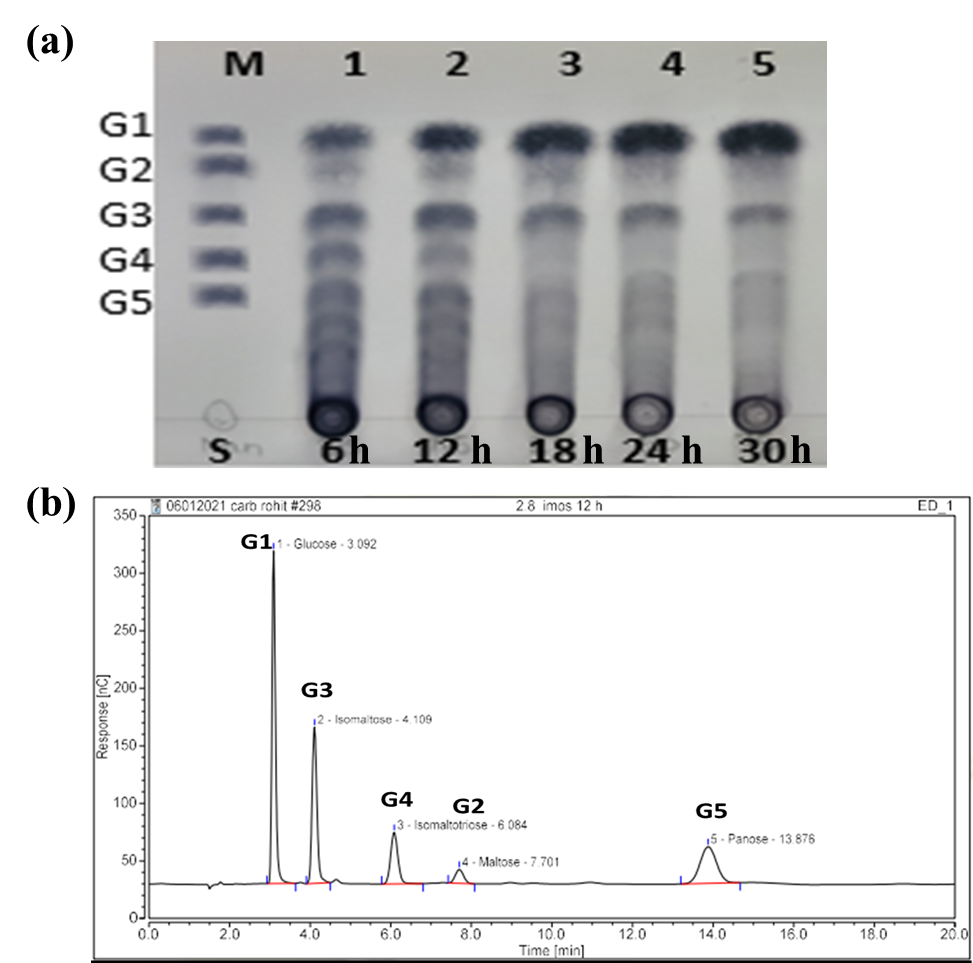


### Figure. S5. Transglucosylation reaction product analysis (A) TLC analysis, lane M, marker. G1, glucose; G2, maltose; G3, isomaltose; G4, isomaltotriose; G5, panose. (B) HPAEC-ED chromatogram analysis, G1, glucose; G2, maltose; G3, isomaltose; G4, isomaltotriose and G5 panose.

**
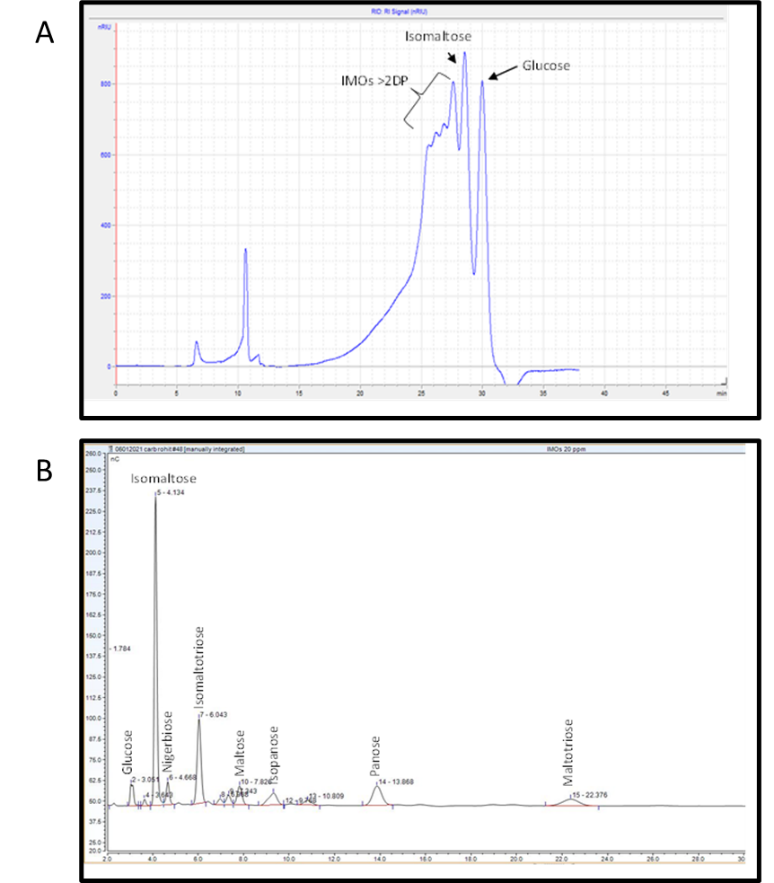
**

**Figure. S6**. SEC purification of IMOs produced by α-transglucosidase: crude IMOs (A); purified IMOs(B).

###

**Figure. S7.** Linkage analysis of PMAA derivative of IMOs.

###
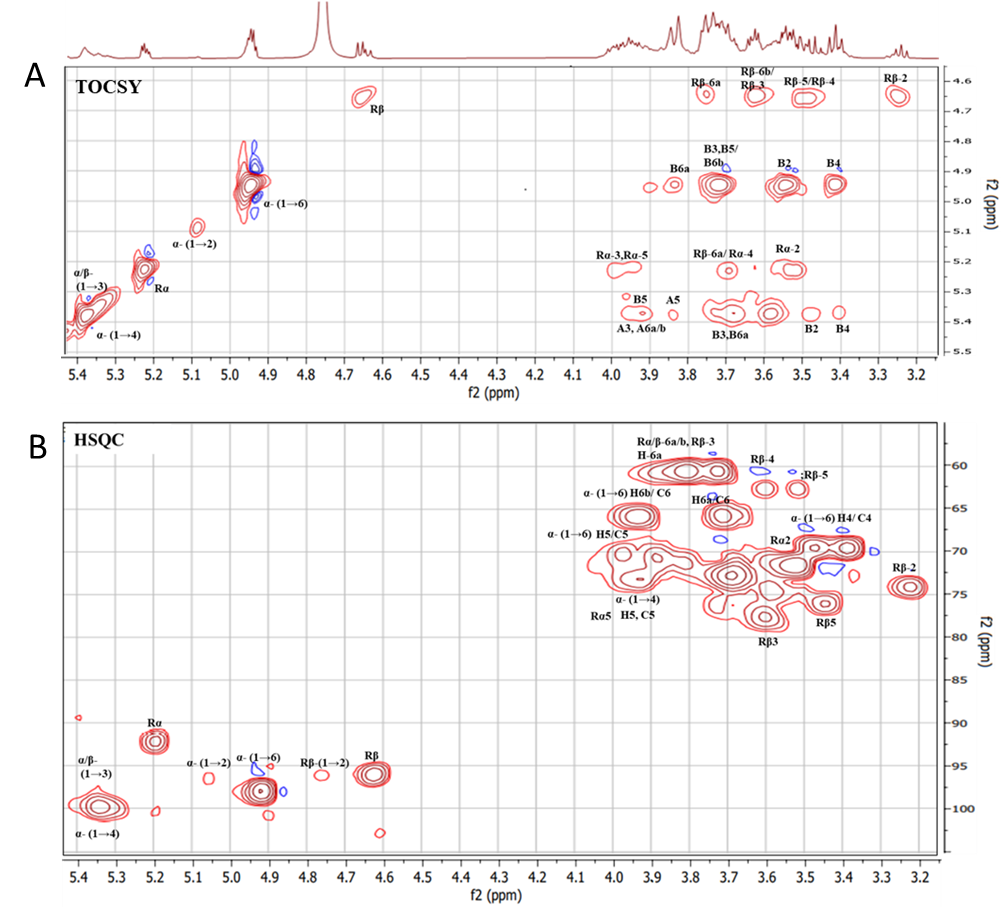


**Figure. S8.** NMR spectrum. **(A)**TOCSY NMR spectrum of IMOs; **(B)** gHSQC NMR spectrum of IMOs.

**Table S1.** ANOVA and model fitting for the response and variables of IMOs

| Source | Sum of Squares | df | Mean Square | F-value | p-value |
| --- | --- | --- | --- | --- | --- |
| Model | 2766.54 | 14 | 197.61 | 33.75 | < 0.0001^**^ |
| A-Enzyme/substrate ratio | 135.34 | 1 | 135.34 | 23.11 | 0.0003^*^ |
| B-Time | 771.20 | 1 | 771.20 | 131.70 | < 0.0001^**^ |
| C-Temperature | 0.0133 | 1 | 0.0133 | 0.0023 | 0.9626 |
| D-pH | 0.1008 | 1 | 0.1008 | 0.0172 | 0.8975 |
| AB | 147.62 | 1 | 147.62 | 25.21 | 0.0002* |
| AC | 105.06 | 1 | 105.06 | 17.94 | 0.0008 |
| AD | 60.06 | 1 | 60.06 | 10.26 | 0.0064 |
| BC | 111.30 | 1 | 111.30 | 19.01 | 0.0007 |
| BD | 17.64 | 1 | 17.64 | 3.01 | 0.1046 |
| CD | 6.25 | 1 | 6.25 | 1.07 | 0.3191 |
| A² | 903.31 | 1 | 903.31 | 154.26 | < 0.0001^**^ |
| B² | 244.41 | 1 | 244.41 | 41.74 | < 0.0001^**^ |
| C² | 459.14 | 1 | 459.14 | 78.41 | < 0.0001^**^ |
| D² | 508.13 | 1 | 508.13 | 86.78 | < 0.0001^**^ |
| Residual | 81.98 | 14 | 5.86 |  |  |
| Pure Error | 3.03 | 4 | 0.7580 |  |  |
| Cor Total | 2848.52 | 28 |  |  |  |

*

*P* < 0.05.

**

*P* < 0.01.
